# Measurement of surface electromyography activity during swallowing in paediatrics: a scoping literature review

**DOI:** 10.1007/s00431-024-05685-2

**Published:** 2024-07-22

**Authors:** Ksenia M. Bykova

**Affiliations:** Rose Centre for Stroke Recovery and Research, School of Psychology Speech and Hearing, University of Canterbury, 249 Papanui Road, Christchurch, 8052 New Zealand

**Keywords:** Paediatrics, Surface electromyography, Neck muscles, Facial muscles, Masticatory muscles, Dysphagia, Deglutition, Cerebral palsy, Infant, Child, Adolescent

## Abstract

Surface electromyography (sEMG) could be used for diagnostic and therapeutic purposes in various health conditions. For example, sEMG biofeedback is shown to be beneficial in adults with swallowing disorders (dysphagia), whereas there are no easily identifiable studies to support such evidence in paediatrics. The current review aimed to evaluate the feasibility of implementing sEMG during swallowing tasks in paediatric populations with various diagnoses. Additionally, the review aimed to describe findings in publications involving participants with cerebral palsy (CP) and dysphagia. Paediatric-related publications were sourced using English keywords and phrases across the following seven databases: PubMed, EMBASE, CINAHL, Web of Science, PsycINFO, and ProQuest Dissertations and Theses Global. The search included all available publications without language and date restrictions. Publications using sEMG during chewing tasks were also accepted in the review as chewing is considered to be part of the act of swallowing. The feasibility of measuring sEMG during swallowing in children with various health conditions was supported by 116 publications (*n* = 6 literature reviews) that met the inclusion criteria for the final full-text review. However, a few publications described some difficulties occurring directly during the sEMG data collection sessions. The review identified 15 publications involving 177 participants with CP who underwent sEMG while swallowing (*n* = 1 publication focused solely on the assessment of chewing). Ten publications described studies that recruited children with dysphagia. Children with CP who had dysphagia were recruited in five of these studies.

*Conclusions*: The acquisition of sEMG measurements while performing swallowing tasks was shown to be feasible in children with various diagnoses including those who have CP and dysphagia. Future studies should investigate the implementation of sEMG as a part of paediatric dysphagia therapy alongside biofeedback swallowing skill training.

**Table:** 

**What is Known:** *• Surface electromyography (sEMG) could be implemented for diagnostic and therapeutic purposes in various health conditions.* *• Biofeedback using sEMG is beneficial in adults with swallowing disorders (dysphagia).* **What is New:** *• Implementation of sEMG was shown to be feasible during swallowing tasks in paediatric populations with various diagnoses, including dysphagia and cerebral palsy.* *• The usage of sEMG biofeedback as a part of paediatric dysphagia management should be investigated in future studies.*

**Supplementary Information:**

The online version contains supplementary material available at 10.1007/s00431-024-05685-2.

## Introduction

Surface electromyography (sEMG) is a widely used neurophysiologic technique for diagnostic and treatment purposes [1]. The sEMG could become a treatment tool when it is incorporated into the biofeedback training of various skills, e.g. swallowing [2, 3] that consists of pre-oral, oral (including chewing), pharyngeal, and oesophageal stages [4]. Currently, there are multiple publications describing the implementation of biofeedback swallowing skill training that resulted in significant improvements in adults with dysphagia (swallowing disorder) who also had diagnoses of stroke [5, 6], Parkinson’s disease [7], motor neurone disease [8], Huntington’s disease [9], multiple system atrophy [10], and in healthy individuals [11, 12]. Since this treatment approach has demonstrated its feasibility in the adult population, it could be considered being applicable to a paediatric population with dysphagia. Especially children with cerebral palsy (CP) could benefit from biofeedback swallowing training since more than half of them have dysphagia and only very few dysphagia management options are available for them with proven effectiveness (e.g., chewing exercises) [13, 14]. A paediatric literature review investigating the feasibility of collecting sEMG measurements while swallowing could be a starting point for a research project on the implementation of swallowing biofeedback training for children.

Three literature reviews [15–17], known to the author, revealed 11 articles focused on research that implemented sEMG during swallowing tasks in a paediatric population that provided preliminary evidence for the feasibility of this approach in children with various diagnoses and encouraged a further search for relevant publications (e.g., in children with CP).

The first systematic literature review aimed to reveal dysphagia screening and evaluation tools in children with neuromuscular diseases (e.g., Duchenne muscular dystrophy (DMD)) [15] and identified only study which used sEMG during swallowing [18].

The second literature review [16] had an aim similar to the one of the current scoping review but focused on research implementing sEMG during feeding only in infants (0–12 months old) and used search strategies that ended in April 2009. Of eight revealed studies, two investigated feeding in preterm infants [19, 20], and six involved term infants [21–25] including one study that recruited infants with Down syndrome [26].

The final systematic literature review [17] searched for studies implementing sEMG in orthodontics. Two publications were identified where the swallowing task [27, 28] was involved.

To continue the search for studies supporting the implementation of biofeedback swallowing training in children, the current scoping literature review will focus on answering the following research questions: (1) What are the diagnoses of children undergoing sEMG during swallowing tasks? (2) Are there studies that have implemented sEMG during swallowing in children with (a) CP and (b) dysphagia? (3) What is the feasibility of implementing sEMG in the paediatric population during swallowing tasks?

## Method

### Eligibility criteria

This scoping literature review had several inclusion criteria: (a) publications should be of the specific types—original articles, narrative and systematic reviews, theses, and conference abstracts and proceedings, (b) reporting a measurement of sEMG activity during swallowing (including breast or bottle feeding and suckling) and chewing (considering chewing is an intrinsic part of the oral stage of swallowing) [4], and (c) having children as participants (including mixed-aged studies and studies where children and adults are distinct samples). The exclusion criteria were (a) publications that were single-case studies (*n* = 1 participant, study with the least generalisable research method) [29], (b) having solely adult participants, and (c) absence of access to a publication’s full-text.

### Information sources and search strategy

After piloting the search syntax, keywords in the English language [(semg OR (surface AND (myograph* OR electromyograph*))) AND (child* OR pediatr* OR infant* OR bab*) AND (swallow* OR dysphag* OR feed*)] (Appendix A) that related to the swallowing function and sEMG implementation in children were used to identify relevant studies. The search was limited only in cases where results showed more than 1000 publications from a search within one database (Appendix A). Seven databases were examined for publication availability in any language through the university library service: PubMed, EMBASE, CINAHL, Scopus, Web of Science, ProQuest Dissertations and Theses Global, and PsycINFO.

Publications mentioned in the reference list of the articles that met the inclusion criteria for the final review were also checked. Additionally, ten experts (researchers) experienced in paediatric sEMG were contacted to request recommendations for relevant publications to be considered for the review.

At the end of the review process, the literature review was updated by checking email alerts used to search for newly published studies from November 16, 2023, to March 31, 2024.

### Data management

EndNote and Rayyan software were used for data management. Citations were uploaded to EndNote to screen them for duplicates and irrelevant publications based on titles. Afterward, the references of unique studies with abstracts and full-text publications were stored on the Rayyan software for systematic reviews. The correctness of translations of non-English publications was verified with native-speaking researchers whenever possible. The full-text publications were obtained when abstracts were determined to meet the inclusion criteria. A table was produced to provide justifications for the exclusion of the full-text publication from the final review (Appendix B), and the PRISMA flow diagram was constructed.

During the final review process of full-text publications, the review matrix [30] was developed for publications related to children with CP or dysphagia. Data was collected for demographic characteristics (age and diagnosis), focus muscles for sEMG including the tasks performed and textures swallowed, and main results. The total sample of full-text publications that met the inclusion criteria was organised in the review matrix containing data about participants’ diagnoses, their residing countries, and muscles assessed during sEMG (Appendix C).

## Results

**Fig. 1:**
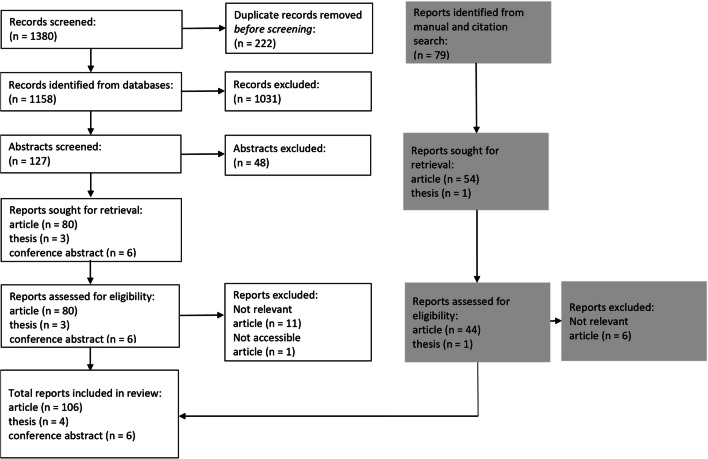
The process of publications review

Following the search of seven databases, the selection process resulted in 1380 studies (Fig. 1). After the removal of duplicates and the exclusion of unrelated studies based on title, 127 abstracts were screened for inclusion/exclusion criteria. The remaining 89 publications were then reviewed, and 12 of them were excluded. Forty-five publications were identified from manual and citation search, and six were excluded. Ineligible studies (*n* = 18) are listed in Appendix B. The literature review resulted in 116 publications (*n* = 9 non-English articles) including six literature reviews, four theses, and six conference proceedings to be incorporated into the review matrix (Appendix C).

### What are the diagnoses of children undergoing surface electromyography during swallowing tasks?

One hundred and ten publications (Appendix C) (plus six literature reviews) related to the implementation of sEMG during swallowing or chewing were included in the final full-text review. Among 110 publications there were 24 publications on healthy children, including nine studies with term infants as participants, and 84 publications that studied children with various diagnoses. These included prematurity (*n* = 8), ankyloglossia (*n* = 3), lip and palate cleft (*n* = 9), tonsillitis (*n* = 1), Down syndrome (*n* = 2), spinal muscular atrophy (*n* = 1), DMD (*n* = 2), dependence on mechanical ventilation (*n* = 1), teeth malocclusion (*n* = 37) (and two publications that specifically focused on incompetent lips and mouth breathing without teeth malocclusion), atypical swallowing (*n* = 4), CP (*n* = 15), and dysphagia as a primary diagnosis (*n* = 1). The chewing task (without swallowing mentioned) was performed in 16 studies out of 110 publications in healthy children (*n* = 4) and children with CP (*n* = 1), malocclusion (*n* = 8), mouth breathing (*n* = 1), and cleft lip and palate (*n* = 2) (Appendix C).

### Are there studies that implement surface electromyography during swallowing in children with cerebral palsy?

Fifteen papers described 11 research projects involving children with CP who underwent sEMG during swallowing (Table 1). The project showed that sEMG was used as a tool to (1) carry out a biofeedback therapy to manage drooling [31], (2) assess muscle state during swallowing [31–35] or chewing [36], and (3) identify swallows during pressure or ultrasound examination of the oral stage of swallowing, respiratory inductance plethysmography, and the establishment of consumed water limit [37–41]. Three research projects (*n* = 5 publications) specified that children with CP also had dysphagia [33, 35, 41].

**Table 1:** Studies measuring surface electromyography (sEMG) activity in children with cerebral palsy (CP)

Author	Group	Muscles	Tasks	Main significant results
Sochaniwskyj et al. (1986) [34]	*N*=36 *n*=24, CP (*n*=12 drooling, *n*=12, non-drooling) *n*=12, TD Age: 6–19 years	- masseter - orbicularis oris - infrahyoid	Swallow (spontaneous saliva, 5 ml and 75 ml juice)	Children with drooling vs. TD & non-drooling had inefficient (not coordinated) and infrequent (45% of TD) swallowing.
Koheil et al. (1987) [31]	*N*=12, CP and drooling Age: 6–18 years	- orbicularis oris - infrahyoid	- Saliva swallow; - Lips puckering and approximation, sucking, and blowing with electrodes placed only on orbicularis oris.	sEMG was used as a swallow assessment and as a part of auditory biofeedback swallow training. There was a significant decrease in drooling.
Kenny et al. (1989) [38]	*N*=7 *n*=4, spastic CP *n*=3, TD Age: 5–12 years	- masseter - infrahyoid	Swallow (5 ml of liquid and a bite-size cookie)	sEMG was used for swallow detection to analyse ultrasound and RIP data. Children with CP had a posterior tongue inactive during swallowing and a slow anterior displacement of the hyoid bone.
McPherson et al. (1992) [40]	*N*=33 *n*=22, CP (*n*=11 spastic, *n*=11, athetoid) *n*=11, TD Age: 4–12 years	- masseter - infrahyoid	Swallow (5 ml and 75 ml of chocolate milk and a bite-size cookie)	sEMG was used for swallow detection to analyse RIP data. Children with CP vs. TD more frequently had multiple swallows with liquids and inspirations at the end of liquid tasks.
Lespargot et al. (1993) [39]	*N*=30 *n*=20, CP (*n*=10 drooling, *n*=10 non-drooling) *n*=10, TD Age: 6–14 years	- submental - infrahyoid	Suck and transport syrup placed under the tongue (1 ml) and behind the lower lip (0.5 ml)	sEMG was used to identify swallows during intraoral pressure measuring and relaxation. Drooling vs. non-drooling children had more frequent multiple swallows.
Casas et al. (1994) [42], Casas (1991) [37]	*N*=40 *n*=20, spastic CP *n*=20, TD Age: 6–12 years	- masseter - infrahyoid	Swallow (5 ml and 75 ml of chocolate milk, and a bite-size cookie)	sEMG was used for swallow detection to analyse RIP data. Children with CP had a greater breathing difficulty with liquids vs. solids boli and large vs. small boli.
Ozdemirkiran et al. (2007) [41]	*N*=40 *n*=12, spastic quadriplegic CP and dysphagia Age: 6–17 years *n*=28, TD Age: 5–16 years	submental	Swallow (3, 5, 8, 12, 15, 20 and 25 ml) – Dysphagia limit test (DLT)	sEMG was used to count swallows. Children with CP had a reduced DLT.
Briesemeister et al. (2013) [36]	*N*=32 *n*=16, spastic CP *n*=16, TD Age: 7–13 years	- masseter - temporalis	Non-habitual mastication	Children with CP had a higher and longer muscle activation (a greater difficulty coordinating masticatory muscles) than TD children.
Tseng et al. (2013) [35]	*N*=80 *n*=20, spastic CP *n*=40, TD Age: 5–30 years, *M*=14.5 years	- submental - infrahyoid	Swallow (3, 5, 8, 12, 15, 20 and 25-ml water). If a 3-ml water bolus is not completed – decrease to 2 and 1 ml.	Subjects with CP vs. TD had a higher maximum sEMG amplitude. Infrahyoids’ maximum amplitude (37.30μV) at 3-ml bolus swallowing showed the highest accuracy to detect dysphagia with sensitivity=85%, specificity=90%, PPV=73.9%, and NPV=94.7%.
Mishra (2017) [33], Mishra et al. (2017) * [43], Mishra et al. (2019) [44]	*N*=21 *n*=11, spastic CP and dysphagia *n*=10, TD Age: 4–11 years	Submental	Swallow (5ml water, 5cc pudding/yogurt, and 1/4 cookie)	Children with spastic CP vs. TD children had 1) neuromuscular overreaction during swallowing pudding/yogurt and cookie, 2) a higher rate of post-swallow inspiration across consistencies (41% vs. 5%).
Malandraki et al. (2022)* [45], Hahn Arkenberg et al. (2023) [32]	*N*=32 *n*=16, unilateral spastic CP *n*=16, TD Age: 7–12 years	- submental - orbicularis oris	Swallow followed by speech: - 5-ml liquid followed by 2 bilabial syllables - 10-ml liquid and 4 bilabial syllables - 5 cc pudding and short sentences	Children with unilateral CP vs. TD children had neuromuscular overactivation during swallowing and speech.
				In children with unilateral CP, it takes longer to reach maximum sEMG amplitude (slower reaction time) vs. TD; and a higher sEMG amplitude in upper lip is associated with shorter mealtime.

*Conference abstract; *CP* cerebral palsy, *TD* typically developing children, *sEMG* surface electromyography, *DLT* Dysphagia limit test, *PPV* positive predictive value, *NPV* negative predictive value, *RIP* respiratory inductance plethysmography

### Are there studies that implement surface electromyography during swallowing in children with dysphagia?

In addition to five publications about children with CP and dysphagia [33, 35, 41, 43, 44], the current scoping literature review identified five more studies involving children with dysphagia (Table 2). Dysphagia also appeared as a comorbidity in children with spinal muscular atrophy [46], DMD [18], brain injury and encephalopathy [47], and prematurity [48]. These five additional studies (Table 2) implemented sEMG as an assessment tool to compare focus groups to healthy participants [18, 46, 47], to evaluate the effect of therapy [47], and to investigate an association of dysphagia with disease severity [49] or submental muscular state with gestational age [48].

**Table 2:** Studies measuring surface electromyography (sEMG) activity in children with dysphagia but without cerebral palsy (CP)

Author	Group	Muscles	Tasks	Main significant results
Van den Engel-Hoek et al. (2009) [46]	*N*=12 *n*=6, spinal muscle atrophy with dysphagia *n*=6, TD Age: 6–13 years	submental	- rest - swallow (5-ml water and 5-ml puree) in usual and forwarded posture	Children with spinal muscle atrophy vs. TD had muscular overreaction which improved in the forwarded body posture
Van den Engel-Hoek et al. (2013)* [50]	*N*=24, DMD with and without dysphagia Age: 6–41 years	submental	Swallow (saliva, 5-ml water, 5-ml custard, 5-ml pure)	The more advance muscular dystrophy stage, the lower sEMG amplitude and harder consumption of pureed food vs. thinner textures
Archer et al. (2013)* [18]	*N*=27, DMD *n*=9, with dysphagia, *n*=6, without dysphagia, *n*=12, TD Age: 16–30 years	- submental - infrahyoid - masseter - orbicularis oris	- maximum voluntary contraction - swallowing (5-ml water)	Subjects with muscular dystrophy and dysphagia vs. TD had muscular overreaction in orbicularis oris and masseters during swallowing, but less activity in submental muscles
He et al. (2019) [47]	*N*=40 *n*=20, dysphagia (brain injury and encephalopathy) *n*=20, TD Age: 3–8 years	- submental - infrahyoid	- rest - swallow (5-ml water)	Children with dysphagia vs. TD had muscular overreaction which improved after one month of SLT
Komisarek et al. (2022) [48]	*N*=16, preterm infants, oral dysphagia Age: 25–32 weeks	- submental - masseter - temporalis - orbicularis oris	Non-nutritive suckling	Younger preterm infants had higher submental muscle tension; the lower umbilic blood pH was associated with the lower muscle tension

^*^mixed age; *TD* typically developing children; *DMD* Duchene muscular dystrophy; *SLT* speech-language therapy targeting dysphagia

### What is the feasibility of implementing sEMG in the paediatric population during swallowing tasks?

Most of the publications reported no issues related to the process of sEMG data obtainment while swallowing. There were a few studies that described the following difficulties that lead to the data loss (number of affected participants, diagnosis): the impossibility of attaching electrodes to the skin (2/15, DMD [18]; 3/12, CP [31]) or recording sEMG signal (5/251, healthy and ankyloglossia [51]; 3/18, healthy infants [25]), detachment of electrodes due to body movements (8/56, healthy infants) [25], interference of external electrical noise (11/251, healthy and ankyloglossia) [51], declining offered liquid (10% glucose) (5/30, healthy infants) [24], participant’s inability to relax (20/41, malocclusion) [52], swallow liquid (spitting it out instead) (1/31, CP) [39] or complete the session (1/19, healthy [32]; 2/23, healthy and CP [33]), and failure to save collected sEMG data (4/38, healthy and CP) [32]. Other issues were evident before the initiation of the sEMG data collection: mandibular anomaly (1/23, malocclusion) [53], challenged intellectual level (1/19, CP) [32], participants missing research visits (not specified (3/52 sessions), healthy [54]; 20/40, ankyloglossia [55]; 10/20, malocclusion [56]; 30/48, healthy infants [25]). One publication merged participants who refused to participate/cooperate, or missed visits into one issue (30/220, tonsillitis) [57].

## Discussion

The current scoping literature review aims to reveal studies supporting the feasibility of implementing sEMG in paediatric populations with variable diagnoses and holds a particular focus on publications involving children with CP and dysphagia. The search was performed for publications in any language without date restrictions and used keywords and phrases in English language. The review resulted in 116 publications (*n* = 6 literature reviews) involving healthy children and children with various health conditions, for example, CP (*n* = 15) and dysphagia as a comorbidity (*n* = 10).

### Surface electromyography across diagnoses

The use of sEMG during swallowing was implemented in children with various diagnoses by applying electrodes to the skin on the following muscles in the neck and/or face (Appendix C): suprahyoid (*n* = 48), infrahyoid (*n* = 18), masseter (*n* = 63), temporal (*n* = 43), perioral (*n* = 44), mentalis (*n* = 11), buccinator (*n* = 4), sternocleidomastoid (*n* = 3), and the dilator naris muscle (*n* = 1). The obtained sEMG data were used to evaluate the effect of intervention (e.g. orthodontic appliances to manage teeth malocclusion), differentiate children with pathological health issues from a healthy population, identify swallowing while also collecting data using alternatives to sEMG (e.g. submental ultrasound), or to describe children with a specific health condition (e.g. CP).

The majority of identified publications included participants with malocclusion (*n* = 37). The investigation of the effect of malocclusion on swallowing could be justified by the possibility that orthodontic disorders could lead to temporomandibular joint dysfunction. That dysfunction could aggravate the biomechanics of the suprahyoid muscles, which are crucial for the oropharyngeal phases of swallowing [4]. The authors evaluating the effect of orthodontic appliances reported either an increase [27, 58–61] or absence of change in the sEMG signal during swallowing [62–65]. Sood et al. [28] suggested that the orthodontic appliances should be used at least for the time required for muscles to return to the baseline sEMG values (the absence of significant difference between pre- and post-treatment timepoints), thereby reflecting an optimal neuromuscular adaptation to the resulted orthodontic changes. However, Zhan et al. [66] stated in the literature review that an increased sEMG activity in masseters after orthodontics treatment is a sign of improved muscle function. In comparison to children with normal teeth occlusion, those with an open bite had lower muscle activity in masseters and temporal muscles during chewing [67, 68] but no difference during swallowing [69]. Children with atypical swallowing also demonstrated a lower intensity of submental muscle activity than healthy children [70]; however, this activity increased after applying an orthodontic device [71] or receiving 10 weeks of myofunctional therapy [72]. Lip incompetence during swallowing manifested in otherwise healthy children as a lower muscle tone in the perioral and temporal muscles [73] but as a higher muscle tone in the perioral muscles of children with Down syndrome [74], malocclusion [53, 75], or atypical swallowing [76].

The literature review identified seven studies with preterm infants, nine studies with children who were born with cleft lip and palate, and four studies with children born with ankyloglossia. Premature infants varied in duration and ratio of sucks to bursts [20] and showed a significant drop in oxygen saturation and ventilation volume during feeding [77]. They swallowed at any point of the respiratory cycle causing, similar to adults, swallowing apneas [78]. In comparison to healthy newborns, preterm infants had more breathing pauses and reached muscle fatigue faster during feeding [79]. The efficient feeders were characterised by the ability to generate a longer sucking burst thereby getting a larger milk volume [19]. When breastfeeding is not possible, cup-feeding (requiring higher involvement of suprahyoid muscles) [80] or bottle-feeding (requiring higher involvement of buccinator) [81] could be considered based on the functionality of the required muscles. Cup-feeding might be difficult for infants born at younger gestational ages associated with more abnormal suprahyoid muscle tone [48].

Children with a repaired cleft lip and palate showed less activity in submental muscles than healthy children during swallowing [82]. However, they demonstrated a higher activity in masseters and temporal muscles during chewing [83] and in infrahyoid [82] and perioral muscles during swallowing [84–87]. Perioral hypertonus did not improve after orthodontic corrections [88, 89]. These abnormal muscle activities reflect a shorter and less efficient oral phase of swallowing and, due to perioral hypertonus, a risk of abnormal craniofacial growth. In children born with a tongue tie (ankyloglossia), the lower activity in submental muscles during breastfeeding was associated with more anterior attachment of the lingual frenulum [51]. Performing a frenectomy resulted in variable muscle responses tested during swallowing and showed either no significant difference in submental muscle activity [55] or its increase to the level of healthy children [90]. The increased submental muscle tone could be required to provide a base of support to the released tongue performing fine movements.

### Surface electromyography in children with cerebral palsy and dysphagia

The literature review process revealed 15 publications (11 studies) involving children with CP as participants. In studies implementing sEMG as an assessment tool, muscular overreaction in infrahyoid and suprahyoid muscle groups while swallowing [32, 33, 35] and in masseters and temporal muscles during chewing [36] was a common sEMG finding. Considering the possible negative impact that hypertonus in the suprahyoid and infrahyoid muscle groups may have on swallowing, sEMG values obtained from infrahyoid muscles proved to be more accurate in detecting dysphagia than the sEMG values obtained from suprahyoid muscles [35]. A high muscle tone in infrahyoid muscles could restrict anterior displacement of the hyoid bone during swallowing by slowing it down as observed in two of the reviewed studies of children with CP [32, 38]. However, this could reflect a delayed swallow initiation due to affected sensory pathways or a slower reaction time. It could be speculated that dysphagia could be ameliorated in children with CP by voluntarily decreasing muscle tone in the anterior neck muscles, especially in the infrahyoid muscle group. In the study of children with dysphagia who underwent swallowing-focused therapy for 1 month, a significant decrease in suprahyoid and infrahyoid muscle tone was observed on sEMG as an identification of successful dysphagia treatment [47]. Higher tone in these muscles might be managed by sEMG biofeedback focused on the relaxation of the upper body since the relaxation of muscles of a smaller submental area is a difficult and time-consuming task for children with CP. In the study by Lespargot et al. [39], only 20% of participants voluntarily managed to relax submental muscles and in the study by Necus [91], two out of three participants were able to complete 30-min relaxation sessions offered twice daily for 6 weeks. Yet in another study, participants with the same diagnosis of CP were able to control muscle tone of a bigger area such as the face [31]. Moreover, children with learning disabilities were able to decrease muscle tension in the arm within ten 10-min sessions [92] resulting in a significant improvement in reading (vs. control group) potentially caused by the relaxation of the muscles adjacent to the arm, e.g. neck and, possibly via platysma, face. The instruction of relaxing upper body parts should be detected by the sEMG electrode attached to the skin of either the suprahyoid or infrahyoid area of the neck. Considering the implementation of sEMG during chewing tasks and the interaction of suprahyoid and masticatory muscles (e.g. contraction of submental muscles during mouth opening) [4], it would be valuable to measure the activity not only of masticatory [36] but also of submental muscles while chewing in future studies.

Regarding swallowing-breathing coordination, children with CP had a higher rate of post-swallow inspirations than healthy children across consistencies [33, 40] that could be caused by a need for extra oxygen after a longer swallowing apnea that occurred earlier, during oral stage of swallowing [38]. The breathing difficulty was significantly more prominent with liquids [37] possibly to protect the lower airways from a preswallow posterior spillage due to an inactive posterior tongue base [38] that is unable to keep the bolus in the oral cavity.

The feasibility of measuring sEMG activity while swallowing in paediatrics was demonstrated in 110 reviewed publications. A few studies reported difficulties occurring during the data collection session, disregarding the health state of participants. The amount of lost data could be decreased in future studies (e.g. on biofeedback) by using familiar food or drink to swallow (milk or formula in neonates), stronger and not allergic adhesive to attach electrodes that are resilient to drooling [32] and considering participants’ ability to follow instructions (intellectual level based on age and severity of disability). Three studies in children with CP (described in six publications) [32, 33, 36, 43–45] considered the intellectual level as one of the eligibility criteria and also specified participants’ level of gross motor skills on the 5-level Gross Motor Function Classification System (GMFCS) [93] as levels I–V (bolus swallowing and chewing tasks) [33, 36, 43, 44] and levels I–II (bolus swallowing and speech tasks) [32, 45]. Participants with CP will probably have sufficient intellectual level to follow instructions (required for biofeedback swallow training) if they attend school and are assigned to levels I–III on the GMFCS [94]. Future studies should investigate how children with CP at GMFCS levels I–III will manage with biofeedback swallow tasks and tolerate the well-adhesive submental sEMG electrode for the length of the session.

The main limitation of this scoping review is the involvement of only one author in the development of the current scoping literature review. That created an increased risk of errors in the literature search and data extraction. The attempts to minimise the risks were the following—obeying the PRISMA guideline [95], using EndNote and Ryyan software, contacting researchers who implemented sEMG in children for the recommendations of relevant publications, and submitting the manuscript to the peer-review journal to get feedback regarding included publications and missed ones, if any. Another limitation is a translation of full-text non-English publications (*n* = 9) without the assistance of licensed translators. Researchers who were Japanese and Chinese native speakers were approached to consult regarding the correctness of the translations of three publications.

In summary, the implementation of sEMG during swallowing tasks was administered in numerous publications that could support the feasibility of this method in the paediatric population with various diagnoses including dysphagia and cerebral palsy. Dysphagia occurs in children with various diagnoses and is mostly associated with sEMG overreaction in the submental or infrahyoid muscles which regulate hyoid and larynx mobility. Considering the child as whole, the muscular hypertonus in the neck muscles might not be present in isolation of the involvement of other body muscles, e.g. shoulders and arms. This could be especially possible in children with brain damage (CP, brain injury) where muscular spasticity is a common state of body muscles. Among children with CP in New Zealand, spastic CP comprises 93% of all cases (New Zealand cerebral palsy report [96]). Therefore, SLP dysphagia therapy might include approaches for decreasing the tone of muscles involved in swallowing such as implementing an sEMG biofeedback method to facilitate voluntary muscle relaxation.

## Supplementary Information

Below are links to the electronic supplementary material.

Appendix A (DOCX 15 KB)

Appendix B (DOCX 33 KB)

Appendix C (DOCX 135 KB)

Supplementary file 1 (DOCX 85 KB)

## Data Availability

No datasets were generated or analysed during the current study.

## Abbreviations

CP: Cerebral palsy
DLT: Dysphagia limit test
DMD: Duchenne muscular dystrophy
NPV: Negative predictive value
PPV: Positive predictive value
RIP: Respiratory inductance plethysmography
sEMG: Surface electromyography
SLT: Speech-language therapy targeting dysphagia
TD: Typically developing children

## References

[1] Alcan V, Zinnuroğlu M (2023) Current developments in surface electromyography. Turk J Med Sci. 2023;53(5):1019-31. 10.55730/1300-0144.5667 10.55730/1300-0144.5667 PMC10763750 38813041

[2] Huckabee M-L, Mills M, Flynn R, Doeltgen S (2023) The evolution of swallowing rehabilitation and emergence of biofeedback modalities. Curr Otorhinol Rep. 11(2):144–53. 10.1007/s40136-023-00451-8

[3] Crider A, Glaros AG, Gevirtz RN (2005) Efficacy of biofeedback-based treatments for temporomandibular disorders. Appl Psychophys Biof. 30:333–45. 10.1007/s10484-005-8420-5 10.1007/s10484-005-8420-5 16385422

[4] Daniels SK, Huckabee ML, Gozdzikowska K (2019) Dysphagia following stroke. 3d ed. San Diego, CA: Plural Publishing, Incorporated; 2019. Available from: https://ebookcentral.proquest.com/lib/canterbury/detail.action?docID=5716560

[5] Loppnow A, Netzebandt J, Frank U, Huckabee M-L (2016) Skill-training in der Dysphagietherapie: Möglichkeiten eines patientenorientierten Vorgehens mittels sEMG-Biofeedback. Spektrum Patholing. 9:243-58. Available from: https://www.researchgate.net/publication/309920525_Skill-Training_in_der_Dysphagietherapie

[6] Burchell D, Hosking S, Kambanaros M, Stiller K (2022) Skill-based swallowing therapy using a computer-based training program improves swallowing-related quality of life and swallowing function for adults with dysphagia: a pilot study. JCPSLP. 24(3):130–7. 10.1080/22087168.2022.12370373

[7] Athukorala RP, Jones RD, Sella O, Huckabee M-L (2014) Skill training for swallowing rehabilitation in patients with Parkinson’s disease. Arch Phys Med Rehabil. 95(7):1374–82. 10.1016/j.apmr.2014.03.001 24816250 10.1016/j.apmr.2014.03.001

[8] Thomas PAL (2020) Impacts of skill training on swallowing and quality of life in patients with motor neurone disease [PhD thesis on the Internet]. Christchurch (NZ): University of Canterbury. 10.26021/10538

[9] Burnip E, Gozdzikowska K, Guiu-Hernandez E, Thomas P, Jury M, Winiker K et al (2021) Skill-based dysphagia training as an intervention for individuals with Huntington’s disease. J Neurol Neurosurg Ps. 92(SUPPL 1):A35. 10.1136/jnnp-2021-EHDN.81

[10] Perry SE, Sevitz JS, Curtis JA, Kuo SH, Troche MS (2018) Skill training resulted in improved swallowing in a person with multiple system atrophy: an endoscopy study. Mov Disord Clin Pract. 5(4):451. 10.1002/mdc3.12628 30838304 10.1002/mdc3.12628 PMC6336184

[11] Erfmann KL, Macrae PR, Jones RD, Guiu Hernandez E, Huckabee M-L (2022) Effects of cerebellar transcranial direct current stimulation (tDCS) on motor skill learning in swallowing. Disabil Rehabil. 44(11):2276–84. 10.1080/09638288.2020.1827303 33001711 10.1080/09638288.2020.1827303

[12] Caruana A, Huckabee M-L, Bradnam L, Doeltgen S (2016) Biofeedback-assisted swallowing skill training: correlation between changes in cortical excitability and swallowing accuracy. Dysphagia. 31(6):797–8. 10.1007/s00455-016-9752-4

[13] Novak I, Morgan C, Fahey M, Finch-Edmondson M, Galea C, Hines A et al (2020) State of the evidence traffic lights 2019: systematic review of interventions for preventing and treating children with cerebral palsy. Curr Neurol Neurosci Rep. 20:1–21. 10.1007/s11910-020-1022-z 32086598 10.1007/s11910-020-1022-z PMC7035308

[14] Speyer R, Cordier R, Kim JH, Cocks N, Michou E, Wilkes-Gillan S (2019) Prevalence of drooling, swallowing, and feeding problems in cerebral palsy across the lifespan: a systematic review and meta-analyses. Dev Med Child Neurol. 61(11):1249–58. 10.1111/dmcn.14316 31328797 10.1111/dmcn.14316

[15] Audag N, Goubau C, Toussaint M, Reychler G (2017) Screening and evaluation tools of dysphagia in children with neuromuscular diseases: a systematic review. Dev Med Child Neurol. 59(6):591–6. 10.1111/dmcn.13354 27935021 10.1111/dmcn.13354

[16] Gomes CF, Thomson Z, Cardoso JR (2009) Utilization of surface electromyography during the feeding of term and preterm infants: a literature review. Dev Med Child Neurol. 51(12):936–42. 10.1111/j.1469-8749.2009.03526.x 19909308 10.1111/j.1469-8749.2009.03526.x

[17] Wozniak K, Piątkowska D, Lipski M, Mehr K (2013) Surface electromyography in orthodontics–a literature review. Med Sci Monit 19:416. 10.12659/MSM.883927 23722255 10.12659/MSM.883927 PMC3673808

[18] Archer SK, Garrod R, Hart N, Miller S (2013) Dysphagia in Duchenne muscular dystrophy assessed objectively by surface electromyography. Dysphagia. 28(2):188–98. 10.1007/s00455-012-9429-6 23179024 10.1007/s00455-012-9429-6

[19] Daniels H, Casaer P, Devlieger H, Eggermont E (1986) Mechanisms of feeding efficiency in preterm infants. J Pediatr Gastroenterol Nutr. 5(4):593–6. 10.1002/j.1536-4801.1986.tb09136.x 3735008 10.1097/00005176-198607000-00015

[20] Nyqvist KH, Färnstrand C, Eeg-Olofsson KE, Ewald U (2001) Early oral behaviour in preterm infants during breastfeeding: an electromyographic study. Acta Paediatr. 90(6):658–63. 10.1111/j.1651-2227.2001.tb02430.x 11440100 10.1080/080352501750258739

[21] Gomes CF, Trezza EMC, Murade ECM, Padovani CR (2006) Surface electromyography of facial muscles during natural and artificial feeding of infants. Jornal de Pediatria. 82(2):103–9. 10.2223/JPED.1456 16614763 10.2223/JPED.1456

[22] Inoue N, Sakashita R, Kamegai T (1995) Reduction of masseter muscle activity in bottle-fed babies. Early Hum Dev. 42(3):185–93. 10.1016/0378-3782(95)01649-N 7493586 10.1016/0378-3782(95)01649-n

[23] Sakashita R, Kamegai T, Inoue N (1996) Masseter muscle activity in bottle feeding with the chewing type bottle teat: evidence from electromyographs. Early Hum Dev. 45(1–2):83–92. 10.1016/0378-3782(96)01723-9 8842642 10.1016/0378-3782(96)01723-9

[24] Tamura Y, Horikawa Y, Yoshida S (1996) Co-ordination of tongue movements and peri-oral muscle activities during nutritive sucking. Dev Med Child Neurol. 38(6):503–10. 10.1111/j.1469-8749.1996.tb12111.x 8647330 10.1111/j.1469-8749.1996.tb12111.x

[25] Tamura Y, Matsushita S, Shinoda K, Yoshida S (1998) Development of perioral muscle activity during suckling in infants: a cross-sectional and follow-up study. Dev Med Child Neurol. 40(5):344–8. 10.1111/j.1469-8749.1998.tb15387.x 9630263

[26] Ideriha PN, Limongi SCO (2007) Electromyographic evaluation of sucking in infants with Down syndrome. Rev Soc Bras Fonoaudiol. 12:174–83. 10.1590/S1516-80342007000300004

[27] Erdem A, Kilic N, Eroz B (2009) Changes in soft tissue profile and electromyographic activity after activator treatment. Aust Orthod J. 25(2):116–22. 10.2478/aoj-2009-0017 20043545

[28] Sood S, Kharbanda O, Duggal R, Sood M, Gulati S (2011) Muscle response during treatment of class II division 1 malocclusion with Forsus fatigue resistant device. J Clin Pediatr Dent 35(3):331–338. Available from: 10.17796/jcpd.35.3.5v86511u4h1mw144 10.17796/jcpd.35.3.5v86511u4h1mw144 21678680

[29] Mariotto FL, Zanni PP, Moraes GHS (2014) What is the use of a single-case study in management research? Rev Admin Empres. 54:358–69. 10.1590/S0034-759020140402

[30] Garrard J (2020) Health sciences literature review made easy. 6^th^ ed. Burlington (US): Jones & Bartlett Learning

[31] Koheil R, Sochaniwskyj AE, Bablich K, Kenny DJ, Milner M (1987) Biofeedback techniques and behaviour modification in the conservative remediation of drooling by children with cerebral palsy. Dev Med Child Neurol. 29(1):19–26. 10.1111/j.1469-8749.1987.tb02103.x 3556797 10.1111/j.1469-8749.1987.tb02103.x

[32] Hahn Arkenberg RE, Mitchell SS, Craig BA, Brown B, Burdo-Hartman W, Lundine JP et al (2023) Neuromuscular adaptations of swallowing and speech in unilateral cerebral palsy: shared and distinctive traits. J Neurophysiol. 130(6):1375–91. 10.1152/jn.00502.2022 37877193 10.1152/jn.00502.2022 PMC11068406

[33] Mishra A (2017) Airway protective behaviors and mealtime performance in children with spastic cerebral palsy and typically developing controls [PhD thesis on the Internet]. New York (US): Columbia University. 10.7916/D8FJ2NF1

[34] Sochaniwskyj AE, Koheil RM, Bablich K, Milner M, Kenny DJ (1986) Oral motor functioning, frequency of swallowing and drooling in normal children and in children with cerebral palsy. Arch Phys Med Rehabil. 67(12):866–74. 10.5555/uri:pii:0003999386900316 3800614

[35] Tseng FF, Tseng SF, Huang YH, Liu CC, Chiang TH (2013) Surface electromyography for diagnosing dysphagia in patients with cerebral palsy. World J Otorhinolaryngol. 3(2):35–41. 10.5319/wjo.v3.i2.35

[36] Briesemeister M, Schmidt KC, Ries LGK (2013) Changes in masticatory muscle activity in children with cerebral palsy. J Electromyogr Kinesiol. 23(1):260–6. 10.1016/j.jelekin.2012.09.002 23063911 10.1016/j.jelekin.2012.09.002

[37] Casas MJ (1991) Ultrasound investigation of ventilation/swallowing interactions during the oral phase of swallow [MSc thesis on the Internet]. University of Toronto, Toronto (CA). Available from: https://www.proquest.com/dissertations-theses/ultrasound-investigation-ventilationswallowing/docview/303982168/se-2

[38] Kenny DJ, Casas MJ, McPherson KA (1989) Correlation of ultrasound imaging of oral swallow with ventilatory alterations in cerebral palsied and normal children: preliminary observations. Dysphagia. 4:112–7. 10.1007/BF02407155 2701093 10.1007/BF02407155

[39] Lespargot A, Langevin MF, Muller S, Guillemont S (1993) Swallowing disturbances associated with drooling in cerebral-palsied children. Dev Med Child Neurol. 35(4):298–304. 10.1111/j.1469-8749.1993.tb11641.x 8335144 10.1111/j.1469-8749.1993.tb11641.x

[40] McPherson KA, Kenny DJ, Koheil R, Bablich K, Sochaniwskyj A, Milner M (1992) Ventilation and swallowing interactions of normal children and children with cerebral palsy. Dev Med Child Neurol. 34(7):577–88. 10.1111/j.1469-8749.1992.tb11488.x 1511793 10.1111/j.1469-8749.1992.tb11488.x

[41] Ozdemirkiran T, Secil Y, Tarlaci S, Ertekin C (2007) An EMG screening method (dysphagia limit) for evaluation of neurogenic dysphagia in childhood above 5 years old. Int J Pediatr Otorhinolaryngol. 71(3):403–7. 10.1016/j.ijporl.2006.11.006 17182111 10.1016/j.ijporl.2006.11.006

[42] Casas MJ, Kenny DJ, McPherson KA (1994) Swallowing/ventilation interactions during oral swallow in normal children and children with cerebral palsy. Dysphagia. 9(1):40–6. 10.1007/BF00262758 8131424 10.1007/BF00262758

[43] Mishra A, Malandraki GA, Sheppard JJ, Gordon AM, Levy E, Troche MS (2017) Airway protective behaviors and clinical swallow function in children with cerebral palsy and healthy controls. Dysphagia. 32(6):801–2. 10.1007/s00455-017-9805-3 10.1007/s00455-018-9933-4 30088088

[44] Mishra A, Malandraki GA, Sheppard JJ, Gordon AM, Levy ES, Troche MS (2019) Voluntary cough and clinical swallow function in children with spastic cerebral palsy and healthy controls. Dysphagia. 34(2):145–54. 10.1007/s00455-018-9933-4 30088088 10.1007/s00455-018-9933-4

[45] Malandraki G, Mitchell S, Arkenberg RH, Brown B, Lundine J, Burdo-Hartman W et al (2022) The neuromuscular control of swallowing and speech in unilateral CP: overactivation and lack of specificity are overlapping traits. Dysphagia. 37(4):1085. 10.1007/s00455-021-10366-5

[46] Van Den Engel-Hoek L, Erasmus CE, Van Bruggen HW, De Swart BJM, Sie LTL, Steenks MH et al (2009) Dysphagia in spinal muscular atrophy type II: more than a bulbar problem? Neurology. 73(21):1787–91. 10.1212/WNL.0b013e3181c34aa6 19933981 10.1212/WNL.0b013e3181c34aa6

[47] He JH, Zhang J, Yuan LP, Qin R, Liu H, Duan YQ et al (2019) Application of surface electromyography in children with dysphagia. Chin J Contemp Pediatr. 21(11):1089–93. 10.7499/j.issn.1008-8830.2019.11.007 10.7499/j.issn.1008-8830.2019.11.007 PMC7389307 31753090

[48] Komisarek O, Malak R, Kwiatkowski J, Wiechec K, Szczapa T, Kasperkowicz J et al (2022) The evaluation of facial muscles by surface electromyography in very preterm infants. Biomed. 10(11):7. 10.3390/biomedicines10112921 10.3390/biomedicines10112921 PMC9687131 36428488

[49] van den Engel-Hoek L, de Groot IJM, Esser E, Gorissen B, Hendriks JCM, de Swart BJM et al (2012) Biomechanical events of swallowing are determined more by bolus consistency than by age or gender. Physiol Behav. 106(2):285–90. 10.1016/j.physbeh.2012.02.018 22369854 10.1016/j.physbeh.2012.02.018

[50] van den Engel-Hoek L, Erasmus CE, Hendriks JC, Geurts AC, Klein WM, Pillen S et al (2013) Oral muscles are progressively affected in Duchenne muscular dystrophy: implications for dysphagia treatment. J Neurol. 260(5):1295–303. 10.1007/s00415-012-6793-y 23263593 10.1007/s00415-012-6793-y

[51] França ECL, Albuquerque LCA, Martinelli RLC, Gonçalves IMF, Souza CB, Barbosa MA (2020) Surface electromyographic analysis of the suprahyoid muscles in infants based on lingual frenulum attachment during breastfeeding. Int J Environ Res Public Health. 17(3):859. 10.3390/ijerph17030859 32019082 10.3390/ijerph17030859 PMC7037214

[52] Harradine N, Kirschen R (1983) Lip and mentalis activity and its influence on incisor position—a quantitative electromyographic study. Br J Orthod. 10(3):114–27. 10.1179/bjo.10.3.114 6575819 10.1179/bjo.10.3.114

[53] Gustafsson M, Ahlgren J (1975) Mentalis and orbicularis oris activity in children with incompetent lips: an electromyographic and cephalometric study. Acta Odontol Scand. 33(6):355–63. 10.3109/00016357509004640

[54] Green JR, Moore CA, Ruark JL, Rodda PR, Morvée WT, Vanwitzenburg MJ (1997) Development of chewing in children from 12 to 48 months: longitudinal study of EMG patterns. J Neurophysiol. 77(5):2704–16. 10.1152/jn.1997.77.5.2704 9163386 10.1152/jn.1997.77.5.2704 PMC3976418

[55] Santos S, da Cunha DA, de Andrade RA, da Silva MG, da Silva Araújo AC, de Castro Martinelli RL et al (2023) Effects of lingual frenotomy on breastfeeding and electrical activity of the masseter and suprahyoid muscles. CODAS. 35(2):e20210262. 10.1590/2317-1782/20232021262 37098939 10.1590/2317-1782/20232021262 PMC10153675

[56] Störmer K, Pancherz H (1999) Electromyography of the perioral and masticatory muscles in orthodontic patients with atypical swallowing. J Orofac Orthop. 60(1):13–23. 10.1007/bf01358712 10028785 10.1007/BF01358712

[57] Vaiman M, Krakovsky D, Eviatar E (2006) The influence of tonsillitis on oral and throat muscles in children. Int J Pediatr Otorhinolaryngol. 70(5):891–8. 10.1016/j.ijporl.2005.09.030 16307800 10.1016/j.ijporl.2005.09.030

[58] Akkaya S, Haydar S, Bilir E (2000) Effects of spring-loaded posterior bite-block appliance on masticatory muscles. Am J Orthod Dentofacial Orthop. 118(2):179–83. 10.1067/mod.2000.104809 10935958 10.1067/mod.2000.104809

[59] Arat FE, Arat ZM, Acar M, Beyazova M, Tompson B (2008) Muscular and condylar response to rapid maxillary expansion, part 1: electromyographic study of anterior temporal and superficial masseter muscles. Am J Orthod Dentofacial Orthop. 133(6):815–22. 10.1016/j.ajodo.2006.07.028 18538244 10.1016/j.ajodo.2006.07.028

[60] Cuevas MJ, Cacho A, Alarcón JA, Martín C (2013) Longitudinal evaluation of jaw muscle activity and mandibular kinematics in young patients with Class II malocclusion treated with the Teuscher activator. Med Oral Patol Oral Cir Bucal. 18(3):e497–e504. 10.4317/medoral.18610 23385506 10.4317/medoral.18610 PMC3668879

[61] De Rossi M, De Rossi A, Hallak JEC, Vitti M, Regalo SCH (2009) Electromyographic evaluation in children having rapid maxillary expansion. Am J Orthod Dentofacial Orthop. 136(3):355–60. 10.1016/j.ajodo.2007.08.027 19732669 10.1016/j.ajodo.2007.08.027

[62] Aggarwal P, Kharbanda OP, Mathur R, Duggal R, Parkash H (1999) Muscle response to the twin-block appliance: an electromyographic study of the masseter and anterior temporal muscles. Am J Orthod Dentofacial Orthop. 116(4):405–14. 10.1016/S0889-5406(99)70225-8 10511668 10.1016/S0889-5406(99)70225-8

[63] Ingervall B, Bitsanis E (1987) A pilot study of the effect of masticatory muscle training on facial growth in long-face children. Eur J Orthod. 9(1):15–23. 10.1093/ejo/9.1.15 3470182 10.1093/ejo/9.1.15

[64] Klocke A, Nanda RS, Ghosh J (2000) Muscle activity with the mandibular lip bumper. Am J Orthod Dentofacial Orthop. 117(4):384–90. 10.1016/S0889-5406(00)70157-0 10756263 10.1016/s0889-5406(00)70157-0

[65] Martín C, Palma JC, Alamán JM, Lopez-Quiñones JM, Alarcón JA (2012) Longitudinal evaluation of sEMG of masticatory muscles and kinematics of mandible changes in children treated for unilateral cross-bite. J Electromyogr Kinesiol. 22(4):620–8. 10.1016/j.jelekin.2012.01.002 22296868 10.1016/j.jelekin.2012.01.002

[66] Zhan Y, Yang M, Bai S, Zhang S, Huang Y, Gong F et al (2023) Effects of orthodontic treatment on masticatory muscles activity: a meta-analysis. Ann Hum Biol. 50(1):465–71. 10.1080/03014460.2023.2271840 37929786 10.1080/03014460.2023.2271840

[67] Ciccone De Faria TDS, Hallak Regalo SC, Thomazinho A, Vitti M, De Felício CM (2010) Masticatory muscle activity in children with a skeletal or dentoalveolar open bite. Eur J Orthod. 32(4):453-8. 10.1093/ejo/cjp132 10.1093/ejo/cjp132 20089569

[68] Piancino MG, Isola G, Merlo A, Dalessandri D, Debernardi C, Bracco P (2012) Chewing pattern and muscular activation in open bite patients. J Electromyogr Kinesiol. 22(2):273–9. 10.1016/j.jelekin.2011.12.003 22236764 10.1016/j.jelekin.2011.12.003

[69] Yousefzadeh F, Shcherbatyy V, King GJ, Huang GJ, Liu ZJ (2010) Cephalometric and electromyographic study of patients of East African ethnicity with and without anterior open bite. Am J Orthod Dentofacial Orthop. 137(2):236–46. 10.1016/j.ajodo.2008.03.033 20152681 10.1016/j.ajodo.2008.03.033

[70] Begnoni G, Cadenas de Llano-Pérula M, Willems G, Pellegrini G, Musto F, Dellavia C (2019) Electromyographic analysis of the oral phase of swallowing in subjects with and without atypical swallowing: a case-control study. J Oral Rehabil. 46(10):927-35. 10.1111/joor.12826 10.1111/joor.12826 31141188

[71] Ciavarella D, Mastrovincenzo M, Sabatucci A, Parziale V, Chimenti C (2010) Effect of the Enveloppe Linguale Nocturne on atypical swallowing: surface electromyography and computerised postural test evaluation. Eur J Paediatr Dent. 11(3):141–5 21080755

[72] Begnoni G, Dellavia C, Pellegrini G, Scarponi L, Schindler A, Pizzorni N (2020) The efficacy of myofunctional therapy in patients with atypical swallowing. Eur Arch Otorhinolaryngol. 277(9):2501–11. 10.1007/s00405-020-05994-w 32367149 10.1007/s00405-020-05994-w

[73] Lipari MA, Pimentel G, Gamboa NA, Bayas I, Guerrero N, Miralles R (2020) Electromyographic comparison of lips and jaw muscles between children with competent and incompetent lips: a cross sectional study. J Clin Pediatr Dent. 44(4):283-7. Available from: https://www.jocpd.com/articles/10.17796/1053-4625-44.4.11 10.17796/1053-4625-44.4.11 33167021

[74] Szyszka-Sommerfeld L, Sycińska-Dziarnowska M, Woźniak K, Machoy M, Wilczyński S, Turkina A et al (2021) The electrical activity of the orbicularis oris muscle in children with down syndrome—a preliminary study. J Clin Med. 10(23):5611. 10.3390/jcm10235611 34884313 10.3390/jcm10235611 PMC8658604

[75] Tosello D, Vitti M, Berzin F (1998) EMG activity of the orbicularis oris and mentalis muscles in children with malocclusion, incompetent lips and atypical swallowing–part I. J Oral Rehabil. 25(11):838–46. 10.1046/j.1365-2842.1998.00322.x 9846904 10.1046/j.1365-2842.1998.00322.x

[76] López-Soto LM, López-Soto OP, Osorio-Forero A, Restrepo F, Tamayo-Orrego L (2017) Muscle activity and muscle strength in atypical swallowing. Revista Salud Uninorte 33(3):273–284. 10.14482/sun.33.3.10890

[77] Timms BJ, DiFiore JM, Martin RJ, Carlo WA, Miller MJ (1992) Alae nasi activation in preterm infants during oral feeding. Pediatr Res. 32(6):679–82. 10.1203/00006450-199212000-00010 1287558 10.1203/00006450-199212000-00010

[78] Wilson SL, Thach BT, Brouillette RT, Abu-Osba YK (1981) Coordination of breathing and swallowing in human infants. J Appl Physiol. 50(4):851–8. 10.1152/jappl.1981.50.4.851 7263368 10.1152/jappl.1981.50.4.851

[79] Hübl N, Riebold B, Schramm D, Seidl RO (2015) Differences in the swallowing process of newborns and healthy preterm infants: first results with a non-invasive bioimpedance and electromyography measurement system. Eur Arch Otorhinolaryngol. 2023:1-12. 10.1007/s00405-023-08344-8 10.1007/s00405-023-08344-8 PMC10796423 37996534

[80] Martins CD, Furlan RMMM, Motta AR, Viana MCFB, editors (2015) Electromyography of muscles involved in feeding premature infants. CoDAS. 27(4):372-7. 10.1590/2317-1782/20152015025 10.1590/2317-1782/20152015025 26398261

[81] Gomes CF, Da Costa Gois MLC, Oliveira BC, Thomson Z, Cardoso JR (2014) Surface electromyography in premature infants: a series of case reports and their methodological aspects. Indian J Pediatr. 81(8):755–9. 10.1007/s12098-013-1199-0 24078289 10.1007/s12098-013-1199-0

[82] Nagaoka K, Tanne K (2007) Activities of the muscles involved in swallowing in patients with cleft lip and palate. Dysphagia. 22(2):140–4. 10.1007/s00455-006-9067-y 17318685 10.1007/s00455-006-9067-y

[83] da Costa LMR, Graciosa MD, Coelho JJ, Rocha R, Ries LGK (2018) Motor behavior of masticatory muscles in individuals with unilateral trans-incisive foramen cleft lip and palate. CRANIO®. 36(4):257-63. 10.1080/08869634.2017.1334021 10.1080/08869634.2017.1334021 28566055

[84] Carvajal R, Miralles R, Cauvi D, Berger B, Carvajal A, Bull R et al (1992) Superior orbicularis oris muscle activity in children with and without cleft lip and palate. Cleft Palate Craniofac J. 29(1):32–7. 10.1597/1545-1569_1992_029_0032_soomai_2.3.co_2 1547249 10.1597/1545-1569_1992_029_0032_soomai_2.3.co_2

[85] Ravera MJ, Miralles R, Santander H, Valenzuela S, Vlllanueva P, Zúñiga C (2000) Comparative study between children with and without cleft lip and cleft palate, part 2: electromyographic analysis. Cleft Palate Craniofac J. 37(3):286–91. 10.1597/1545-1569_2000_037_0286_csbcwa_2.3.co_2 10830809 10.1597/1545-1569_2000_037_0286_csbcwa_2.3.co_2

[86] Szyszka-Sommerfeld L, Machoy ME, Wilczyński S, Lipski M, Woźniak K (2021) Superior orbicularis oris muscle activity in children surgically treated for bilateral complete cleft lip and palate. J Clin Med. 10(8):1720. 10.3390/jcm10081720 33923491 10.3390/jcm10081720 PMC8074006

[87] Szyszka-Sommerfeld L, Woźniak K, Matthews-Brzozowska T, Kawala B, Mikulewicz M (2017) Electromyographic analysis of superior orbicularis oris muscle function in children surgically treated for unilateral complete cleft lip and palate. J Craniomaxillofac Surg. 45(9):1547–51. 10.1016/j.jcms.2017.06.012 28736109 10.1016/j.jcms.2017.06.012

[88] Carvajal R, Miralles R, José Ravera M, Carvajal A, Cauvi D, Manns A (1995) Follow-up of electromyographic and cephalometric findings in patients with unilateral cleft lip and palate after fifteen months of continuous wearing of a special removable appliance. Cleft Palate Craniofac J. 32(4):323–7. 10.1597/1545-1569_1995_032_0323_fuoeac_2.3.co_2 7548106 10.1597/1545-1569_1995_032_0323_fuoeac_2.3.co_2

[89] Carvajal R, Miralles R, Ravera MJ, Cauvi D, Manns A, Carvajal A (1994) Electromyographic and cephalometric findings in patients with unilateral cleft lip and palate after the use of a special removable appliance. Cleft Palate Craniofac J. 31(3):173–8. 10.1597/1545-1569_1994_031_0173_eacfip_2.3.co_2 8068699 10.1597/1545-1569_1994_031_0173_eacfip_2.3.co_2

[90] Tecco S, Baldini A, Mummolo S, Marchetti E, Giuca MR, Marzo G et al (2015) Frenulectomy of the tongue and the influence of rehabilitation exercises on the sEMG activity of masticatory muscles. J Electromyogr Kinesiol. 25(4):619–28. 10.1016/j.jelekin.2015.04.003 25979198 10.1016/j.jelekin.2015.04.003

[91] Necus EF (2011) sEMG biofeedback as a tool to improve oral motor control and functional swallowing in school age children with cerebral palsy: a case series [MSc thesis on the Internet]. Christchurch (NZ): University of Canterbury. 10.26021/7055

[92] Carter JL, Russell HL (1985) Use of EMG biofeedback procedures with learning disabled children in a clinical and an educational setting. J Learn Disabil. 18(4):213–6. 10.1177/002221948501800406 3886819 10.1177/002221948501800406

[93] Bodkin AW, Robinson C, Perales FP (2003) Reliability and validity of the gross motor function classification system for cerebral palsy. Pediatr Phys Ther. 15(4):247–52. 10.1097/01.PEP.0000096384.19136.02 17057460 10.1097/01.PEP.0000096384.19136.02

[94] Delacy MJ, Reid SM, Group ACPR (2016) Profile of associated impairments at age 5 years in Australia by cerebral palsy subtype and Gross Motor Function Classification System level for birth years 1996 to 2005. Dev Med Child Neurol. 58:50–6. 10.1111/dmcn.13012 26777873 10.1111/dmcn.13012

[95] Tricco AC, Lillie E, Zarin W, O’Brien KK, Colquhoun H, Levac D et al (2018) PRISMA extension for scoping reviews (PRISMA-ScR): checklist and explanation. Ann Intern Med. 169(7):467–73. 10.7326/M18-0850 30178033 10.7326/M18-0850

[96] The New Zealand cerebral palsy register report 2022. 2022. Available from: https://nz.cpregister.com/Public/nzl/20220908_NZCPR_Report.pdf

