# Supplementary material for: Measurement of surface electromyography activity during swallowing in paediatrics: a scoping literature review
Source: Eur J Pediatr. 2024 Jul 22;183(10):4145–57. doi: 10.1007/s00431-024-05685-2 (PMC11413118; doi:10.1007/s00431-024-05685-2)
Supplement: Supplementary file 1 — Appendix A (DOCX 15 KB) [file 431_2024_5685_MOESM1_ESM.docx]

**Appendix A**

KEY-WORDS USED FOR THE LITERATURE SEARCH

The last update: March 31, 2024

**Embase, Web of science, PubMed, PsycINFO** :

(semg OR (surface AND (myograph* OR electromyograph*))) AND (child* OR pediatr* OR infant* OR bab*) AND (swallow* OR dysphag* OR feed*)

**Scopus**:

ALL ( ( ( swallow* OR dysphag* OR feed* ) AND ( semg OR "surface myograph*" OR "surface electromyograph*" ) AND ( child* OR pediatr* OR infant* OR bab* ) ) ) AND ( LIMIT-TO ( EXACTKEYWORD , "Child" ) OR LIMIT-TO ( EXACTKEYWORD , "Adolescent" ) OR LIMIT-TO ( EXACTKEYWORD , "School Child" ) OR LIMIT-TO ( EXACTKEYWORD , "Preschool Child" ) OR LIMIT-TO ( EXACTKEYWORD , "Infant" ) OR LIMIT-TO ( EXACTKEYWORD , "Child, Preschool" ) )

**ProQuest Dissertations & Theses:**

Limited to: ("Electromyography" OR "Children" OR "Biofeedback" OR "Cerebral palsy" OR "Swallowing" OR "Dysphagia" OR "EMG" OR "Neurofeedback")

((semg OR (surface AND (myograph* OR electromyograph*))) AND (child* OR pediatr* OR infant* OR bab*) AND (swallow* OR dysphag* OR feed*)) AND diskw.exact("Electromyography" OR "Children" OR "Biofeedback" OR "Cerebral palsy" OR "Swallowing" OR "Dysphagia" OR "EMG" OR "Neurofeedback")
