# Supplementary material for: Measurement of surface electromyography activity during swallowing in paediatrics: a scoping literature review
Source: Eur J Pediatr. 2024 Jul 22;183(10):4145–57. doi: 10.1007/s00431-024-05685-2 (PMC11413118; doi:10.1007/s00431-024-05685-2)
Supplement: Supplementary file 2 — Appendix B (DOCX 33 KB) [file 431_2024_5685_MOESM2_ESM.docx]

**Appendix B**

LIST OF EXCLUDED PUBLICATIONS FROM THE FINAL FULL-TEXT REVIEW

| **Study** | **Reasons for exclusion** |
| --- | --- |
| Achmad et al. (2020) (1) | Study that did not have a swallowing task. |
| Busanello-Stella et al. (2015) (2) | Study that did not have a swallowing task. |
| De Felicio et al. (2016) (3) | Study that did not have a swallowing task. |
| Hafstrom et al. (1997) (4) | Study that did not have a swallowing task. |
| Lenguas et al. (2012) (5) | Study that did not have a swallowing task. |
| Messina et al. (2019) (6) | Study that did not implement sEMG but a myoscan while using an sEMG sensor. |
| Necka, Kawala, & Mattews-Brzozowska (2007) (7) | There is no access to the full-text publication. |
| Necus (2011) (8) | Study that did not have a swallowing task. |
| O’Dwyer & Nicholas (1988) (9) | Study that has adults as participants. |
| Ortu et al. (2019) (10) | Study that did not have a swallowing task. |
| Park (2016) (11) | Study that did not have a swallowing task. |
| Remijn et al. (2018) (12) | Study that did not have a swallowing task. |
| Renault & Raimbault (1992) (13) | Study that did not implement sEMG but needle EMG. |
| Schmidt et al. (1991) (14) | Study that did not have a swallowing task. |
| Szyszka-Sommerfeld et al. (2022) (15) | Study that did not have a swallowing task. |
| Tosello, Vitti, & Berzin (1999) (16) | Study that did not have a swallowing task. |
| Wozniak et al. (2015) (17) | Study that did not have a swallowing task. |
| Yilmaz (1997) (18) | Study that did not have a swallowing task. |

*sEMG* – surface electromyography, *EMG* – electromyography.

References:

1. Achmad H, Areni IS, Mutmainnah N, Inayah NH, Hidayat M, Ramadhany YF. Detection of temporomandibular muscle disorders in children. development of android-based electromyography tools(EMG dentosmart). Systematic Reviews in Pharmacy. 2020;11(6):129-35.

2. Busanello-Stella AR, Blanco-Dutra AP, Corrêa ECR, da Silva AMT. Electromyographic fatigue of orbicular oris muscles during exercises in mouth and nasal breathing children. CODAS. 2015;27(1):80-8.

3. de Felício CM, da Silva Dias FV, Folha GA, de Almeida LA, de Souza JF, Anselmo-Lima WT, et al. Orofacial motor functions in pediatric obstructive sleep apnea and implications for myofunctional therapy. International Journal of Pediatric Otorhinolaryngology. 2016;90:5-11.

4. Hafström M, Lundquist C, Lindecrantz K, Larsson K, Kjellmer I. Recording non‐nutritive sucking in the neonate. Description of an automatized system for analysis. Acta Paediatrica. 1997;86(1):82-90.

5. Lenguas L, Alarcón JA, Venancio F, Kassem M, Martín C. Surface electromyographic evaluation of jaw muscles in children with unilateral crossbite and lateral shift in the early mixed dentition. Sexual dimorphism. Medicina Oral, Patologia Oral y Cirugia Bucal. 2012;17(6):e1096-e102.

6. Messina G, Giustino V, Martines F, Rizzo S, Pirino A, Scoppa F. Orofacial muscles activity in children with swallowing dysfunction and removable functional appliances. Eur J Transl Myol. 2019;29(3):246-50.

7. Nęcka A, Kawala B, Matthews-Brzozowska T. Evaluation of facial muscle tone using surface electromyography in children with Down’s syndrome. Ann Acad Med Stetin. 2007;53:98-101.

8. Necus EF. sEMG biofeedback as a tool to improve oral motor control and functional swallowing in school age children with cerebral palsy: a case series. 2011.

9. O'Dwyer NJ. Control of speech muscles in normal and cerebral-palsied speakers: an electromyographic analysis: UNSW Sydney; 1988.

10. Ortu E, Pietropaoli D, Adib F, Masci C, Giannoni M, Monaco A. Electromyographic evaluation in children orthodontically treated for skeletal Class II malocclusion: Comparison of two treatment techniques. Cranio - Journal of Craniomandibular Practice. 2019;37(2):129-35.

11. Park J. Genioglossus EMG Activity in Normal and Sleep Breathing Disorder Patients [M.S.]. United States -- Maryland: University of Maryland, Baltimore; 2016.

12. Remijn L, Vermaire JA, Nijhuis-van de Sanden MWG, Groen BE, Speksnijder CM. Validity and reliability of the mixing ability test as masticatory performance outcome in children with spastic cerebral palsy and children with typical development: A pilot study. Journal of Oral Rehabilitation. 2018;45(10):790-7.

13. Renault F, Raimbault J. Electromyographie faciale, linguale et pharyngée chez l'enfant: une méthode d'étude des troubles de succion-déglutition et de leur physiopathologie. Neurophysiologie Clinique/Clinical Neurophysiology. 1992;22(3):249-60.

14. Schmidt R, Jonas I, Schulte-Mönting J, Kappert HF, Rakosi T. Integrated and frequency spectra of the electromyograms in patients with temporomandibular symptoms. Fortschritte der Kieferorthopädie. 1991;52(6):339-45.

15. Szyszka-Sommerfeld L, Sycińska-Dziarnowska M, Machoy M, Wilczyński S, Maglitto M, Cernera M, et al. Electromyographic Study of Masticatory Muscle Function in Children with Down Syndrome. Journal of Clinical Medicine. 2022;11(3).

16. Tosello D, Vitti M, Berzin F. EMG activity of the orbicularis oris and mentalis muscles in children with malocclusion, incompetent lips and atypical swallowing–part I. Journal of oral rehabilitation. 1998;25(11):838-46.

17. Woźniak K, Piątkowska D, Szyszka-Sommerfeld L, Buczkowska-Radlińska J. Impact of functional appliances on muscle activity: A surface electromyography study in children. Medical Science Monitor. 2015;21:246-53.

18. Yılmaz L. Masseter Ve ön Temporal kasların Aktivitelerinin Erken Süt dişi Ka- Yıplarında Ve Yer Tutucu Uygulaması Sonrasında Elektromyografik Olarak Incelenmesi [Ph.D.]. Turkey: Marmara Universitesi (Turkey); 1997.
