# Supplementary material for: Measurement of surface electromyography activity during swallowing in paediatrics: a scoping literature review
Source: Eur J Pediatr. 2024 Jul 22;183(10):4145–57. doi: 10.1007/s00431-024-05685-2 (PMC11413118; doi:10.1007/s00431-024-05685-2)
Supplement: Supplementary file 3 — Appendix C (DOCX 135 KB) [file 431_2024_5685_MOESM3_ESM.docx]

**Appendix C.**

STUDIES MEASURING SURFACE ELECTROMYOGRAPHY ACTIVITY DURING SWALLOWING (INCLUDING CHEWING) IN CHILDREN

| **Diagnoses and signs** | **Countries** | **Authors** | **SHM** | **IHM** | **MM** | **TM** | **OO** | **BM** | **Scm** | **Me** | **AN** |
| --- | --- | --- | --- | --- | --- | --- | --- | --- | --- | --- | --- |
| Cerebral palsy | Canada | Sochaniwskyj et al. (1986)(1) |  | x | x |  | x |  |  |  |  |
|  | Canada | Koheil et al. (1987)(2) |  | x |  |  | x |  |  |  |  |
|  | Canada | Kenny et al. (1989)(3) |  | x | x |  |  |  |  |  |  |
|  | Canada | Casas (1991)(4) |  | x | x |  |  |  |  |  |  |
|  | Canada | McPherson et al. (1992)(5) |  | x | x |  |  |  |  |  |  |
|  | France | Lespargot et al. (1993)(6) | x | x |  |  |  |  |  |  |  |
|  | Canada | Casas et al. (1994)(7) |  | x | x |  |  |  |  |  |  |
| with dysphagia | Turkey | Ozdemirkiran et al. (2007)(8) | x |  |  |  |  |  |  |  |  |
|  | Brazil | Briesemeister et al. (2013)^a^(9) |  |  | x | x |  |  |  |  |  |
| with dysphagia | Taiwan | Tseng et al. (2013)(10) | x | x |  |  |  |  |  |  |  |
| with dysphagia | USA | Mishra (2017)^b^(11) | x |  |  |  |  |  |  |  |  |
| with dysphagia | USA | Mishra et al. (2017)(12) | x |  |  |  |  |  |  |  |  |
| with dysphagia | USA | Mishra et al. (2019)(13) | x |  |  |  |  |  |  |  |  |
|  | USA | Malandraki et al. (2022)^b^(14) | x |  |  |  | x |  |  |  |  |
|  | USA | Hahn Arkenberg et al. (2023)(15) | x |  |  |  | x |  |  |  |  |
|  |  |  |  |  |  |  |  |  |  |  |  |
| Dysphagia  brain injury and encephalopathy | China | He et al. (2019)(16) | x | x |  |  |  |  |  |  |  |
|  |  |  |  |  |  |  |  |  |  |  |  |
| Down syndrome | Brazil | Ideriha & Limongi (2007)(17) |  |  |  |  | x |  |  |  |  |
|  | Poland | Szyszka-Sommerfeld et al. (2021)(18) |  |  |  |  | x |  |  |  |  |
|  |  |  |  |  |  |  |  |  |  |  |  |
| Spinal muscular atrophy  with **dysphagia** | Netherlands | Van den Engel-Hoek et al. (2009)(19) | x |  |  |  |  |  |  |  |  |
|  |  |  |  |  |  |  |  |  |  |  |  |
| Duchenne muscular dystrophy  with and without **dysphagia** | Netherlands | Van den Engel-Hoek et al. (2013)(20) | x |  |  |  |  |  |  |  |  |
|  | UK | Archer et al. (2012)(21) | x | x | x |  | x |  |  |  |  |
|  |  |  |  |  |  |  |  |  |  |  |  |
| Teeth malocclusion | Sweden | Ingervall et al. (1975)(22) |  |  | x | x |  |  |  |  |  |
|  | Sweden | Pancherz et al. (1978)^a^(23) |  |  | x | x |  |  |  |  |  |
|  | Sweden | Gustafsson et al. (1975)(24) |  |  | x |  | x |  |  | x |  |
|  | Sweden | Pancherz et al. (1980)^a^(25) |  |  | x | x |  |  |  |  |  |
|  | UK | Harradine et al. (1983)(26) |  |  | x |  | x |  |  | x |  |
|  | Canada | Lowe & Takada (1984)(27) |  |  | x | x | x |  |  |  |  |
|  | Switzerland | Ingervall & Bitsanis (1987)^a^(28) |  |  | x | x |  |  |  |  |  |
|  | Chile | Miralles et al. (1988)(29) |  |  | x | x |  |  |  |  |  |
|  | France | Santana Penin et al. (1992)(30) | x |  | x | x |  |  |  |  |  |
|  | Sweden | Stavridi & Ahlgren (1992)(31) |  |  | x |  |  | x |  | x |  |
|  | Sweden | Ahlgren (1995)(32) |  |  | x |  | x | x |  | x |  |
|  | Brazil | Tosello et al. (1998)(33) |  |  |  |  | x |  |  | x |  |
|  | Germany | Störmer & Pancherz, 1999(34) |  |  | x | x | x |  |  | x |  |
|  | Turkey | Akkaya et al. (2000)(35) |  |  | x | x |  |  |  |  |  |
|  | USA | Klocke et al. (2000)(36) |  |  |  |  | x |  |  |  |  |
|  | Spain | Alarcon et al. (2000)(37) | x |  | x | x |  |  |  |  |  |
|  | Brazil | Vieira et al. (2005)(38) |  |  |  |  | x |  |  | x |  |
|  | Turkey | Arat et al. (2008)(39) |  |  | x | x |  |  |  |  |  |
|  | Turkey | Erdem et al. (2009)(40) |  |  | x | x | x |  |  |  |  |
|  | Brazil | De Rossi et al. (2009)^a^(41) |  |  | x | x |  |  |  |  |  |
|  | Brazil | Ambrosio et al. (2009)(42) |  |  |  |  | x |  |  |  |  |
|  | Brazil | Ambrosio et al. (2009)(43) |  |  |  |  | x |  |  |  |  |
|  | Brazil | Andrade et al. (2010)^a^(44) |  |  | x | x |  |  |  |  |  |
|  | Brazil | Ciccone de Faria et al. (2010)^a^(45) |  |  | x | x |  |  |  |  |  |
|  | USA | Yousefzadeh et al. (2010)(46) | x |  | x | x | x |  |  |  |  |
|  | Italy | Saccucci et al. (2011)(47) |  |  |  |  | x |  |  |  |  |
|  | India | Sood et al. (2011)(48) |  |  | x | x |  |  |  |  |  |
|  | Italy | Piancino et al. (2012)^a^(49) |  |  | x | x |  |  |  |  |  |
|  | Spain | Martin et al. (2012)(50) | x |  | x | x |  |  |  |  |  |
|  | India | Aggarwal et al. (2013)(51) |  |  | x | x |  |  |  |  |  |
|  | Spain | Cuevas et al. (2013)(52) | x |  | x | x |  |  |  |  |  |
|  | USA | Elliot et al. (2015)(53) | x |  | x |  |  |  |  |  |  |
|  | Italy | Piancino et al. (2016)^a^(54) |  |  | x |  |  |  |  |  |  |
|  | Italy | Nucci et al. (2021)(55) |  |  |  |  | x |  |  |  |  |
|  | Spain | Garcia et al. (2022)(56) | x |  | x | x | x |  |  |  |  |
|  | China | Zhao et al. (2023)(57) |  |  | x | x | x |  |  | x |  |
|  | Bulgaria | Bogdanov et al. (2023)(58) |  |  |  |  | x |  |  | x |  |
|  |  |  |  |  |  |  |  |  |  |  |  |
| Incompetent lips | Chile | Lipari et al. (2020)(59) |  |  |  | x | x |  |  |  |  |
| Mouth breathing | Brazil | Ferla et al. (2008)^a^(60) |  |  | x | x |  |  |  |  |  |
|  |  |  |  |  |  |  |  |  |  |  |  |
| on AVL^c^  sepsis, airway and heart diseases | Brazil | Amantea et al. (2004)(61) |  | x |  |  |  |  |  |  |  |
|  |  |  |  |  |  |  |  |  |  |  |  |
| Lip and palate cleft | Chile | Carvajal et al. (1992)(62) |  |  |  |  | x |  |  |  |  |
|  | Chile | Carvajal et al. (1994)(63) |  |  |  |  | x |  |  |  |  |
|  | Chile | Carvajal et al. (1995)(64) |  |  |  |  | x |  |  |  |  |
|  | Chile | Ravera et al. (2000)(65) |  |  |  |  | x |  |  |  |  |
|  | Japan | Nagaoka et al. (2007)(66) | x | x |  |  |  |  |  |  |  |
|  | Poland | Szyszka-Sommerfeld et al. (2017)(67) |  |  |  |  | x |  |  |  |  |
|  | Brazil | Da Costa et al. (2018)^a^(68) |  |  | x | x |  |  |  |  |  |
|  | Brazil | Sabbag et al. (2018)^a^(69) |  |  | x | x |  |  |  |  |  |
|  | Poland | Szyszka-Sommerfeld et al. (2021)(70) |  |  |  |  | x |  |  |  |  |
|  |  |  |  |  |  |  |  |  |  |  |  |
| Atypical swallowing | Italy | Ciavarella et al. (2010)(71) |  |  | x | x |  |  | x |  |  |
|  | Colombia | Lopez-Soto et al. (2017)(72) |  |  | x |  | x |  |  | x |  |
|  | Italy & Belgium | Begnoni et al. (2019)(73) | x |  | x | x |  |  |  |  |  |
|  | Italy | Begnoni et al. (2020)(74) | x |  | x | x |  |  |  |  |  |
|  |  |  |  |  |  |  |  |  |  |  |  |
| Ankyloglossia | Italy | Tecco et al. (2015)(75) | x |  | x | x | x |  |  |  |  |
|  | Brazil | Franca et al. (2020)(76) | x |  |  |  |  |  |  |  |  |
|  | Brazil | Santos et al. (2023)(77) | x |  | x |  |  |  |  |  |  |
|  |  |  |  |  |  |  |  |  |  |  |  |
| Tonsillitis | Israel | Vaiman et al. (2006)(78) | x | x | x |  |  |  |  |  |  |
|  |  |  |  |  |  |  |  |  |  |  |  |
| Prematurity | USA | Wilson et al. (1981)(79) | x |  |  |  |  |  |  |  |  |
|  | Belgium | Daniels et al. (1986)(80) | x |  |  |  |  |  |  |  |  |
|  | USA | Timms et al. (1992)^b^(81) |  |  |  |  |  |  |  |  | x |
|  | Sweden | Nyqvist et al. (2001)(82) | x | x |  |  | x |  |  |  |  |
|  | Brazil | Gomes et al. (2014)(83) |  |  | x |  |  | x |  |  |  |
|  | Brazil | Martins et al. (2015)(84) | x |  | x | x |  |  |  |  |  |
| with **feeding difficulties** | Poland | Komisarek et al. (2022)(85) | x |  | x | x | x |  |  |  |  |
|  | Germany | Hubl et al. (2023)(86) | x |  |  |  |  |  | x |  |  |
|  |  |  |  |  |  |  |  |  |  |  |  |
| Healthy | Sweden | Pancherz (1980)^a^(87) |  |  | x | x |  |  |  |  |  |
|  | Japan | Takarada et al. (1990)^a^(88) |  |  | x | x |  |  |  |  |  |
|  | Japan | Inoue et al. (1995)(89) |  |  | x |  |  |  |  |  |  |
|  | Japan | Sakashita et al. (1996)(90) |  |  | x |  |  |  |  |  |  |
|  | Japan | Tamura et al. (1996)(91) | x |  | x | x | x |  |  |  |  |
|  | USA | Green et al. (1997)^a^(92) | x |  | x | x |  |  |  |  |  |
|  | USA | Ruark & Moore (1997)^a^(93) | x | x |  |  | x |  |  |  |  |
|  | Japan | Tamura et al. (1998)(94) | x |  | x | x | x |  |  |  |  |
|  | USA | Ruark et al. (2002)(95) | x | x |  |  | x |  |  |  |  |
|  | Israel | Vaiman et al. (2004)(96) | x | x | x |  |  |  |  |  |  |
|  | Brazil | Jacinto-Goncalves et al. (2005)(97) |  |  |  |  | x |  |  | x |  |
|  | Japan | Sato & Nakashima (2007)(98) | x | x |  |  |  |  |  |  |  |
|  | Netherlands | Van den Engel-Hoek et al. (2012)(99) | x |  |  |  |  |  |  |  |  |
|  | USA | Hahn Arkenberg et al. (2022)^b^(100) | x |  |  |  |  |  |  |  |  |
|  | USA | Hahn Arkenberg et al. (2023)(101) | x |  |  |  | x |  |  |  |  |
|  |  |  |  |  |  |  |  |  |  |  |  |
| Healthy (mature, applicable to infants) | Japan | Tamura et al. (1992)(102) | x |  | x | x | x |  |  |  |  |
|  | Japan | Sou et al. (1994)(103) | x |  | x | x | x |  |  |  |  |
|  | Japan | Matsushita et al. (1994)(104) | x |  | x | x | x |  |  |  |  |
|  | Brazil | Gomes et al. (2006)(105) |  |  | x | x |  | x |  |  |  |
|  | Israel | Ratnovsky et al. (2013)(106) | x |  |  |  | x |  | x |  |  |
|  | Brazil | Franca et al. (2014)(107) |  |  | x |  |  |  |  |  |  |
|  | Netherlands | Lagarde et al. (2018)^b^(108) | x |  |  |  |  |  |  |  |  |
|  | Netherlands | Lagarde et al. (2019)^b^(109) | x |  |  |  |  |  |  |  |  |
|  | Netherlands | Lagarde et al. (2019) (110) | x |  |  |  |  |  |  |  |  |

^a^ – studies that used chewing task during surface electromyography without swallowing mentioned,

^b^ - conference proceeding,

^c^ - AVL – artificial ventilation of the lungs

SHM – suprahyoid muscles, IHM – infrahyoid muscles, MM – masseters, TM – temporal muscles, BM – buccinators, OO – orbicularis oris, Scm – sternocleidmastoids, Me – mentalis, AN – alae nasi.

References:

1. Sochaniwskyj AE, Koheil RM, Bablich K, Milner M, Kenny DJ. Oral motor functioning, frequency of swallowing and drooling in normal children and in children with cerebral palsy. Arch Phys Med Rehabil. 1986;67(12):866-74.

2. Koheil R, Sochaniwskyj AE, Bablich K, Kenny DJ, Milner M. Biofeedback techniques and behaviour modification in the conservative remediation of drooling by children with cerebral palsy. Developmental Medicine & Child Neurology. 1987;29(1):19-26.

3. Kenny DJ, Casas MJ, McPherson KA. Correlation of ultrasound imaging of oral swallow with ventilatory alterations in cerebral palsied and normal children: preliminary observations. Dysphagia. 1989;4:112-7.

4. Casas MJ. Ultrasound investigation of ventilation/swallowing interactions during the oral phase of swallow: University of Toronto; 1994.

5. McPherson KA, Kenny DJ, Koheil R, Bablich K, Sochaniwskyj A, Milner M. Ventilation and swallowing interactions of normal children and children with cerebral palsy. Developmental Medicine & Child Neurology. 1992;34(7):577-88.

6. Lespargot A, Langevin MF, Muller S, Guillemont S. Swallowing disturbances associated with drooling in cerebral‐palsied children. Developmental Medicine & Child Neurology. 1993;35(4):298-304.

7. Casas MJ, Kenny DJ, McPherson KA. Swallowing/ventilation interactions during oral swallow in normal children and children with cerebral palsy. Dysphagia. 1994;9(1):40-6.

8. Ozdemirkiran T, Secil Y, Tarlaci S, Ertekin C. An EMG screening method (dysphagia limit) for evaluation of neurogenic dysphagia in childhood above 5 years old. Int J Pediatr Otorhinolaryngol. 2007;71(3):403-7.

9. Briesemeister M, Schmidt KC, Ries LGK. Changes in masticatory muscle activity in children with cerebral palsy. Journal of Electromyography and Kinesiology. 2013;23(1):260-6.

10. Tseng F-F, Tseng S-F, Huang Y-H, Liu C-C, Chiang T-H. Surface electromyography for diagnosing dysphagia in patients with cerebral palsy. World Journal of Otorhinolaryngology. 2013;3(2):35-41.

11. Mishra A. Airway protective behaviors and mealtime performance in children with spastic cerebral palsy and typically developing controls [Ph.D.]. United States -- New York: Columbia University; 2017.

12. Mishra A, Malandraki GA, Sheppard JJ, Gordon AM, Levy E, Troche MS. Airway protective behaviors and clinical swallow function in children with cerebral palsy and healthy controls. Dysphagia. 2017;32(6):801-2.

13. Mishra A, Malandraki GA, Sheppard JJ, Gordon AM, Levy ES, Troche MS. Voluntary Cough and Clinical Swallow Function in Children with Spastic Cerebral Palsy and Healthy Controls. Dysphagia. 2019;34(2):145-54.

14. Malandraki GA, Mitchell SS, Hahn Arkenberg RE, Brown B, Craig BΑ, Burdo-Hartman W, et al. Swallowing and motor speech skills in unilateral cerebral palsy: Novel findings from a preliminary cross-sectional study. Journal of Speech, Language, and Hearing Research. 2022;65(9):3300-15.

15. Hahn Arkenberg RE, Mitchell SS, Craig BA, Brown B, Burdo-Hartman W, Lundine JP, et al. Neuromuscular adaptations of swallowing and speech in Unilateral Cerebral Palsy: shared and distinctive traits. Journal of Neurophysiology. 2023.

16. He JH, Zhang J, Yuan LP, Qin R, Liu H, Duan YQ, et al. [Application of surface electromyography in children with dysphagia]. Zhongguo dang dai er ke za zhi = Chinese journal of contemporary pediatrics. 2019;21(11):1089-93.

17. Ideriha PN, Limongi SCO. Electromyographic evaluation of sucking in infants with Down syndrome. Revista da Sociedade Brasileira de Fonoaudiologia. 2007;12:174-83.

18. Szyszka-Sommerfeld L, Sycińska-Dziarnowska M, Woźniak K, Machoy M, Wilczyński S, Turkina A, et al. The electrical activity of the orbicularis oris muscle in children with down syndrome—a preliminary study. Journal of Clinical Medicine. 2021;10(23).

19. Van Den Engel-Hoek L, Erasmus CE, Van Bruggen HW, De Swart BJM, Sie LTL, Steenks MH, et al. Dysphagia in spinal muscular atrophy type II: More than a bulbar problem? Neurology. 2009;73(21):1787-91.

20. van den Engel-Hoek L, Erasmus CE, Hendriks JC, Geurts AC, Klein WM, Pillen S, et al. Oral muscles are progressively affected in Duchenne muscular dystrophy: implications for dysphagia treatment. J Neurol. 2013;260(5):1295-303.

21. Archer SK, Garrod R, Hart N, Miller S. Dysphagia in Duchenne muscular dystrophy assessed objectively by surface electromyography. Dysphagia. 2013;28(2):188-98.

22. Ingervall B, Thilander B. Activity of temporal and masseter muscles in children with a lateral forced bite. The Angle orthodontist. 1975;45(4):249-58.

23. Pancherz H, Anehus M. Masticatory function after activator treatment: An analysis of masticatory efficiency, occlusal contact conditions and EMG activity. Acta Odontologica Scandinavica. 1978;36(5-6):309-16.

24. Gustafsson M, Ahlgren J. Mentalis and orbicularis oris activity in children with incompetent lips: an electromyographic and cephalometric study. Acta Odontologica Scandinavica. 1975;33(6):355-63.

25. Pancherz H, Anehus-Pancherz M. Muscle activity in Class II, Division 1 malocclusions treated by bite jumping with the Herbst appliance: an electromyographic study. American journal of orthodontics. 1980;78(3):321-9.

26. Harradine N, Kirschen R. Lip and mentalis activity and its influence on incisor position—a quantitative electromyographic study. British journal of orthodontics. 1983;10(3):114-27.

27. Lowe AA, Takada K. Associations between anterior temporal, masseter, and orbicularis oris muscle activity and craniofacial morphology in children. American journal of orthodontics. 1984;86(4):319-30.

28. Ingervall B, Bitsanis E. A pilot study of the effect of masticatory muscle training on facial growth in long-face children. The European Journal of Orthodontics. 1987;9(1):15-23.

29. Miralles R, Berger B, Bull R, Manns A, Carvajal R. Influence of the activator on electromyographic activity of mandibular elevator muscles. Am J Orthod Dentofacial Orthop. 1988;94(2):97-103.

30. Santana Penin UA, Mora Bermúdez MJ, Fraga Bermúdez JM, Raymond JL. [Anomalies of the inclination of the occlusal plane: early treatment using indirect guide-planes--electromyographic response]. Orthod Fr. 1992;63 Pt 2:619-33.

31. Stavridi R, Ahlgren J. Muscle response to the oral-screen activator. An EMG study of the masseter, buccinator, and mentalis muscles. The European Journal of Orthodontics. 1992;14(5):339-49.

32. Ahlgren J. EMG studies of lip and cheek activity in sucking habits. Swedish dental journal. 1995;19(3):95-101.

33. Tosello DO, Vitti M, Berzin F. EMG activity of the orbicularis oris and mentalis muscles in children with malocclusion, incompetent lips and atypical swallowing--part II. Journal of oral rehabilitation. 1999;26(8):644-9.

34. Störmer K, Pancherz H. Electromyography of the perioral and masticatory muscles in orthodontic patients with atypical swallowing. Journal of Orofacial Orthopedics= Fortschritte der Kieferorthopadie: Organ/official Journal Deutsche Gesellschaft fur Kieferorthopadie. 1999;60(1):13-23.

35. Akkaya S, Haydar S, Bilir E. Effects of spring-loaded posterior bite-block appliance on masticatory muscles. American Journal of Orthodontics and Dentofacial Orthopedics. 2000;118(2):179-83.

36. Klocke A, Nanda RS, Ghosh J. Muscle activity with the mandibular lip bumper. American Journal of Orthodontics & Dentofacial Orthopedics. 2000;117(4):384-90.

37. Alarcón JA, Martín C, Palma JC. Effect of unilateral posterior crossbite on the electromyographic activity of human masticatory muscles. American journal of orthodontics and dentofacial orthopedics : official publication of the American Association of Orthodontists, its constituent societies, and the American Board of Orthodontics. 2000;118(3):328-34.

38. Vieira SW, Tanaka OM, Maruo H, Essenfelder LRC, Vieira S. A perioral muscles activity, buccal-lingual inclination and space discrepancy of the lower incisors study, in mouth or nasal breathing children with normal or Class I malocclusion. Revista Dental Press de Ortodontia e Ortopedia Facial. 2005;10:108-16.

39. Arat FE, Arat ZM, Acar M, Beyazova M, Tompson B. Muscular and condylar response to rapid maxillary expansion. Part 1: Electromyographic study of anterior temporal and superficial masseter muscles. American Journal of Orthodontics and Dentofacial Orthopedics. 2008;133(6):815-22.

40. Erdem A, Kilic N, Eroz B. Changes in soft tissue profile and electromyographic activity after activator treatment. Australian Orthodontic Journal. 2009;25(2):116-22.

41. De Rossi M, De Rossi A, Hallak JEC, Vitti M, Regalo SCH. Electromyographic evaluation in children having rapid maxillary expansion. American Journal of Orthodontics and Dentofacial Orthopedics. 2009;136(3):355-60.

42. Ambrosio AR, Trevilatto PC, Martins LP, dos Santos-Pinto A, Shimizu RH. Electromyographic evaluation of the upper lip according to the breathing mode: A longitudinal study. Brazilian Oral Research. 2009;23(4):415-23.

43. Ambrosio AR, Trevilatto PC, Sakima T, Ignácio SA, Shimizu RH. Correlation between morphology and function of the upper lip: A longitudinal evaluation. European Journal of Orthodontics. 2009;31(3):306-13.

44. Andrade AdS, Gavião MBD, Gameiro GH, Rossi MD. Characteristics of masticatory muscles in children with unilateral posterior crossbite. Brazilian Oral Research. 2010;24:204-10.

45. Ciccone De Faria TDS, Hallak Regalo SC, Thomazinho A, Vitti M, De Felício CM. Masticatory muscle activity in children with a skeletal or dentoalveolar open bite. European Journal of Orthodontics. 2010;32(4):453-8.

46. Yousefzadeh F, Shcherbatyy V, King GJ, Huang GJ, Liu ZJ. Cephalometric and electromyographic study of patients of East African ethnicity with and without anterior open bite. American Journal of Orthodontics and Dentofacial Orthopedics. 2010;137(2):236-46.

47. Saccucci M, Tecco S, Ierardoa G, Luzzi V, Festa F, Polimeni A. Effects of interceptive orthodontics on orbicular muscle activity: A surface electromyographic study in children. Journal of Electromyography and Kinesiology. 2011;21(4):665-71.

48. Sood S, Kharbanda O, Duggal R, Sood M, Gulati S. Muscle response during treatment of Class II division 1 malocclusion with Forsus fatigue resistant device. Journal of Clinical Pediatric Dentistry. 2011;35(3):331-8.

49. Piancino MG, Isola G, Merlo A, Dalessandri D, Debernardi C, Bracco P. Chewing pattern and muscular activation in open bite patients. Journal of Electromyography and Kinesiology. 2012;22(2):273-9.

50. Martín C, Palma JC, Alamán JM, Lopez-Quiñones JM, Alarcón JA. Longitudinal evaluation of sEMG of masticatory muscles and kinematics of mandible changes in children treated for unilateral cross-bite. Journal of Electromyography and Kinesiology. 2012;22(4):620-8.

51. Aggarwal P, Kharbanda OP, Mathur R, Duggal R, Parkash H. Muscle response to the twin-block appliance: an electromyographic study of the masseter and anterior temporal muscles. American journal of orthodontics and dentofacial orthopedics : official publication of the American Association of Orthodontists, its constituent societies, and the American Board of Orthodontics. 1999;116(4):405-14.

52. Cuevas MJ, Cacho A, Alarcón JA, Martín C. Longitudinal evaluation of jaw muscle activity and mandibular kinematics in young patients with Class II malocclusion treated with the Teuscher activator. Medicina Oral, Patologia Oral y Cirugia Bucal. 2013;18(3):e497-e504.

53. Elliott S. Instrumental Investigation of the Effects of Tongue Thrust on Swallow Function [M.Sc.]. United States -- Idaho: Idaho State University; 2016.

54. Piancino MG, Falla D, Merlo A, Vallelonga T, De Biase C, Dalessandri D, et al. Effects of therapy on masseter activity and chewing kinematics in patients with unilateral posterior crossbite. Archives of Oral Biology. 2016;67:61-7.

55. Nucci L, Marra PM, Femiano L, Isola G, Flores-Mir C, Perillo L, et al. Perioral muscle activity changes after Lip Bumper treatment. European Journal of Paediatric Dentistry. 2021;22(2):129-34.

56. Garcia AEJ, Clari VR, Gallardo VP. Relationship between demographic and cephalometric measures and electromyographic activity of the facial musculature. A preliminary study in children and adolescents. Rev Investig Logop. 2022;12(1):10.

57. Zhao M, Han M, Habumugisha J, Mohamed AS, Bu W, Guo Y, et al. Electromyographic activities of the jaw and facial muscles in subjects with different vertical skeletal patterns and breathing modes. Journal of Oral Rehabilitation. 2023;50(5):351-9.

58. Bogdanov V, Dinkova M, Tsakova D. Effect of pre-orthodontic trainer treatment on the masticatory and perioral muscles in patients with class II subdivision 1 malocclusion evaluated by surface EMG. Folia Medica. 2023;65(5):816-20.

59. Lipari MA, Pimentel G, Gamboa NA, Bayas I, Guerrero N, Miralles R. Electromyographic Comparison of Lips and Jaw Muscles between Children With Competent and Incompetent Lips: A Cross Sectional Study. Journal of Clinical Pediatric Dentistry. 2020;44(4):283-7.

60. Ferla A, Silva AMTd, Corrêa ECR. Electrical activity of the anterior temporal and masseter muscles in mouth and nasal breathing children. Revista Brasileira de Otorrinolaringologia. 2008;74:588-95.

61. Amantéa SL, Piva JP, Sanches PR, Palombini BC. Oropharyngeal aspiration in pediatric patients with endotracheal intubation. Pediatric critical care medicine : a journal of the Society of Critical Care Medicine and the World Federation of Pediatric Intensive and Critical Care Societies. 2004;5(2):152-6.

62. Carvajal R, Miralles R, Cauvi D, Berger B, Carvajal A, Bull R, et al. Superior orbicularis oris muscle activity in children with and without cleft lip and palate. Cleft Palate-Craniofacial Journal. 1992;29(1):32-7.

63. Carvajal R, Miralles R, Ravera MJ, Cauvi D, Manns A, Carvajal A. Electromyographic and cephalometric findings in patients with unilateral cleft lip and palate after the use of a special removable appliance. The Cleft palate-craniofacial journal. 1994;31(3):173-8.

64. Carvajal R, Miralles R, José Ravera M, Carvajal A, Cauvi D, Manns A. Follow-up of electromyographic and cephalometric findings in patients with unilateral cleft lip and palate after fifteen months of continuous wearing of a special removable appliance. The Cleft palate-craniofacial journal. 1995;32(4):323-7.

65. Ravera MJ, Miralles R, Santander H, Valenzuela S, Vlllanueva P, Zúñiga C. Comparative study between children with and without cleft lip and cleft palate, Part 2: Electromyographic analysis. Cleft Palate-Craniofacial Journal. 2000;37(3):286-91.

66. Nagaoka K, Tanne K. Activities of the muscles involved in swallowing in patients with cleft lip and palate. Dysphagia. 2007;22(2):140-4.

67. Szyszka-Sommerfeld L, Woźniak K, Matthews-Brzozowska T, Kawala B, Mikulewicz M. Electromyographic analysis of superior orbicularis oris muscle function in children surgically treated for unilateral complete cleft lip and palate. Journal of cranio-maxillo-facial surgery : official publication of the European Association for Cranio-Maxillo-Facial Surgery. 2017;45(9):1547-51.

68. da Costa LMR, Graciosa MD, Coelho JJ, Rocha R, Ries LGK. Motor behavior of masticatory muscles in individuals with unilateral trans-incisive foramen cleft lip and palate. CRANIO®. 2018;36(4):257-63.

69. Sabbag A, Denadai R, Raposo-Amaral CA, Buzzo CL, Raposo-Amaral CE, Nagae MH. Electromyographic activity of the masseter and temporal muscles in patients with nonsyndromic complete unilateral cleft lip and palate: 2-stage versus 1-stage palate repair. Journal of Craniofacial Surgery. 2018;29(6):1463-8.

70. Szyszka-Sommerfeld L, Machoy ME, Wilczyński S, Lipski M, Woźniak K. Superior orbicularis oris muscle activity in children surgically treated for bilateral complete cleft lip and palate. Journal of Clinical Medicine. 2021;10(8).

71. Ciavarella D, Mastrovincenzo M, Sabatucci A, Parziale V, Chimenti C. Effect of the Enveloppe Lingua le Nocturne on atypical swallowing: surface electromyography and computerised postural test evaluation. European Journal of Paediatric Dentistry. 2010;11(3):141-5.

72. López-Soto LM, López-Soto OP, Osorio-Forero A, Restrepo F, Tamayo-Orrego L. Muscle Activity and Muscle Strength in Atypical Swallowing. Revista Salud Uninorte. 2017;33(3):273-84.

73. Begnoni G, Cadenas de Llano-Pérula M, Willems G, Pellegrini G, Musto F, Dellavia C. Electromyographic analysis of the oral phase of swallowing in subjects with and without atypical swallowing: A case-control study. Journal of oral rehabilitation. 2019;46(10):927-35.

74. Begnoni G, Dellavia C, Pellegrini G, Scarponi L, Schindler A, Pizzorni N. The efficacy of myofunctional therapy in patients with atypical swallowing. European Archives of Oto-Rhino-Laryngology. 2020;277(9):2501-11.

75. Tecco S, Baldini A, Mummolo S, Marchetti E, Giuca MR, Marzo G, et al. Frenulectomy of the tongue and the influence of rehabilitation exercises on the sEMG activity of masticatory muscles. Journal of Electromyography and Kinesiology. 2015;25(4):619-28.

76. França ECL, Albuquerque LCA, Martinelli RLC, Gonçalves IMF, Souza CB, Barbosa MA. Surface Electromyographic Analysis of the Suprahyoid Muscles in Infants Based on Lingual Frenulum Attachment during Breastfeeding. International journal of environmental research and public health. 2020;17(3).

77. Santos S, da Cunha DA, de Andrade RA, da Silva MG, da Silva Araújo AC, de Castro Martinelli RL, et al. Effects of lingual frenotomy on breastfeeding and electrical activity of the masseter and suprahyoid muscles. CODAS. 2023;35(2).

78. Vaiman M, Krakovsky D, Eviatar E. The influence of tonsillitis on oral and throat muscles in children. International Journal of Pediatric Otorhinolaryngology. 2006;70(5):891-8.

79. Wilson SL, Thach BT, Brouillette RT, Abu-Osba YK. Coordination of breathing and swallowing in human infants. Journal of Applied Physiology. 1981;50(4):851-8.

80. Daniels H, Casaer P, Devlieger H, Eggermont E. Mechanisms of feeding efficiency in preterm infants. Journal of pediatric gastroenterology and nutrition. 1986;5(4):593-6.

81. Timms BJ, DiFiore JM, Martin RJ, Carlo WA, Miller MJ. Alae nasi activation in preterm infants during oral feeding. Pediatric research. 1992;32(6):679-82.

82. Nyqvist KH, Färnstrand C, Eeg-Olofsson KE, Ewald U. Early oral behaviour in preterm infants during breastfeeding: an electromyographic study. Acta paediatrica (Oslo, Norway : 1992). 2001;90(6):658-63.

83. Gomes CF, Da Costa Gois MLC, Oliveira BC, Thomson Z, Cardoso JR. Surface electromyography in premature infants: A series of case reports and their methodological aspects. Indian Journal of Pediatrics. 2014;81(8):755-9.

84. Martins CD, Furlan RMMM, Motta AR, Viana MCFB, editors. Electromyography of muscles involved in feeding premature infants. CoDAS; 2015: SciELO Brasil.

85. Komisarek O, Malak R, Kwiatkowski J, Wiechec K, Szczapa T, Kasperkowicz J, et al. The Evaluation of Facial Muscles by Surface Electromyography in Very Preterm Infants. Biomedicines. 2022;10(11):7.

86. Hübl N, Riebold B, Schramm D, Seidl RO. Differences in the swallowing process of newborns and healthy preterm infants: first results with a non-invasive bioimpedance and electromyography measurement system. European Archives of Oto-Rhino-Laryngology. 2023:1-12.

87. Pancherz H. Temporal and masseter muscle activity in children and adults with normal occlusion An electromyographic investigation. Acta Odontologica Scandinavica. 1980;38(6):343-8.

88. Takarada T, Larrinaga GA, Nishida F, Nishino M. Frequency analyses of EMG power spectra of anterior temporal and masseter muscles in children and adults. Dentistry in Japan. 1990;27(1):119-25.

89. Inoue N, Sakashita R, Kamegai T. Reduction of masseter muscle activity in bottle-fed babies. Early human development. 1995;42(3):185-93.

90. Sakashita R, Kamegai T, Inoue N. Masseter muscle activity in bottle feeding with the chewing type bottle teat: evidence from electromyographs. Early Human Development. 1996;45(1-2):83-92.

91. Tamura Y, Horikawa Y, Yoshida S. Co‐ordination of tongue movements and peri‐oral muscle activities during nutritive sucking. Developmental Medicine & Child Neurology. 1996;38(6):503-10.

92. Green JR, Moore CA, Ruark JL, Rodda PR, Morvée WT, Vanwitzenburg MJ. Development of chewing in children from 12 to 48 months: Longitudinal study of EMG patterns. Journal of Neurophysiology. 1997;77(5):2704-16.

93. Ruark JL, Moore CA. Coordination of lip muscle activity by 2-year-old children during speech and nonspeech tasks. Journal of Speech, Language, and Hearing Research. 1997;40(6):1373-85.

94. Tamura Y, Matsushita S, Shinoda K, Yoshida S. Development of perioral muscle activity during suckling in infants: a cross‐sectional and follow‐up study. Developmental Medicine & Child Neurology. 1998;40(5):344-8.

95. Ruark JL, McCullough GH, Peters RL, Moore CA. Bolus consistency and swallowing in children and adults. Dysphagia. 2002;17:24-33.

96. Vaiman M, Segal S, Eviatar E. Surface electromyographic studies of swallowing in normal children, age 4-12 years. International Journal of Pediatric Otorhinolaryngology. 2004;68(1):65-73.

97. Jacinto-Gonçalves SR, Gavião MBD, Berzin F, De Oliveira AS, Semeguini TA. Electromyographic activity of perioral muscle in breastfed and non-breastfed children. Journal of Clinical Pediatric Dentistry. 2004;29(1):57-62.

98. Sato K, Nakashima T. Sleep-related deglutition in children. Annals of Otology, Rhinology and Laryngology. 2007;116(10):747-53.

99. van den Engel-Hoek L, de Groot IJM, Esser E, Gorissen B, Hendriks JCM, de Swart BJM, et al. Biomechanical events of swallowing are determined more by bolus consistency than by age or gender. Physiol Behav. 2012;106(2):285-90.

100. Hahn Arkenberg RE, Mitchell S, Brown B, Goffman L, Malandraki G. The Neuromuscular Development of Swallowing Continues into the School-Age Years: Evidence from a Preliminary Cross-sectional Study. Dysphagia. 2022;37(4):1084.

101. Hahn Arkenberg RE, Brown B, Mitchell S, Craig BΑ, Goffman L, Malandraki GA. Shared and Separate Neuromuscular Underpinnings of Swallowing and Motor Speech Development in the School-Age Years. Journal of speech, language, and hearing research : JSLHR. 2023:1-16.

102. Tamura Y, Sou M, Narita Y, Mishima T. Masticatory Muscle Activities During Sucking, 1. Coordination between sucking movements and masticatory muscles. Jpn J Ped Dentistry. 1992;30(1):150-7.

103. Sou M. Masticatory muscle activities during sucking, 2. Differences between breast and bottle feeding. Jpn J Ped Dent. 1992;30:541-50.

104. Matsushita S, Horikawa Y, Tamura Y, Yoshida S. Changes of lectromyographic activities of perioral muscles during sucking periods in infants. Jpn J Pediatr Dent. 1994;32(4):817-25.

105. Gomes CF, Trezza EMC, Murade ECM, Padovani CR. Surface electromyography of facial muscles during natural and artificial feeding of infants. Jornal de Pediatria. 2006;82(2):103-9.

106. Ratnovsky A, Carmeli YN, Elad D, Zaretsky U, Dollberg S, Mandel D. Analysis of facial and inspiratory muscles performance during breastfeeding. Technol Health Care. 2013;21(5):511-20.

107. França ECL, Sousa CB, Aragão LC, Costa LR. Electromyographic analysis of masseter muscle in newborns during suction in breast, bottle or cup feeding. BMC Pregnancy and Childbirth. 2014;14(1).

108. Lagarde MLJ, Van Alfen N, De Groot SAF, Van Den Engel-Hoek L. Coordination of sucking, swallowing and breathing during nutritive sucking in healthy infants. Dysphagia. 2018;33(4):571.

109. Lagarde MLJ, Van Alfen N, De Groot SAF, Geurts ACH, Van Den Engel-Hoek L. Adaptation of 2-5 months old infants to flow shape and flexibility of teats during bottle feeding. Dysphagia. 2019;34(5):720-1.

110. Lagarde M, van Alfen N, De Groot S, Geurts A, van den Engel-Hoek L. Adaptive capacity of 2-to 5-month-old infants to the flow, shape, and flexibility of different teats during bottle feeding: a cross-sectional study. BMC pediatrics. 2019;19:1-7.
